# High Throughput Sequencing for Detection of Foodborne Pathogens

**DOI:** 10.3389/fmicb.2017.02029

**Published:** 2017-10-20

**Authors:** Camilla Sekse, Arne Holst-Jensen, Ulrich Dobrindt, Gro S. Johannessen, Weihua Li, Bjørn Spilsberg, Jianxin Shi

**Affiliations:** ^1^Department of Animal Health and Food Safety, Norwegian Veterinary Institute, Oslo, Norway; ^2^Institute of Hygiene, University of Münster, Münster, Germany; ^3^Joint International Research Laboratory of Metabolic and Developmental Sciences, Shanghai Jiao Tong University–University of Adelaide Joint Centre for Agriculture and Health, School of Life Sciences and Biotechnology, Shanghai Jiao Tong University, Shanghai, China; ^4^Department of Analysis and Diagnostics, Norwegian Veterinary Institute, Oslo, Norway

**Keywords:** bacteria and viruses, fungi and parasites, metagenomics, microbial profiling, outbreak investigation, surveillance, metataxonomics, whole genome sequencing

## Abstract

High-throughput sequencing (HTS) is becoming the state-of-the-art technology for typing of microbial isolates, especially in clinical samples. Yet, its application is still in its infancy for monitoring and outbreak investigations of foods. Here we review the published literature, covering not only bacterial but also viral and Eukaryote food pathogens, to assess the status and potential of HTS implementation to inform stakeholders, improve food safety and reduce outbreak impacts. The developments in sequencing technology and bioinformatics have outpaced the capacity to analyze and interpret the sequence data. The influence of sample processing, nucleic acid extraction and purification, harmonized protocols for generation and interpretation of data, and properly annotated and curated reference databases including non-pathogenic “natural” strains are other major obstacles to the realization of the full potential of HTS in analytical food surveillance, epidemiological and outbreak investigations, and in complementing preventive approaches for the control and management of foodborne pathogens. Despite significant obstacles, the achieved progress in capacity and broadening of the application range over the last decade is impressive and unprecedented, as illustrated with the chosen examples from the literature. Large consortia, often with broad international participation, are making coordinated efforts to cope with many of the mentioned obstacles. Further rapid progress can therefore be prospected for the next decade.

## Introduction

### Foodborne pathogens and their impact

Foodborne pathogens (FBPs) cause foodborne diseases (FBDs) either directly (by infectious agents) or indirectly (by toxic metabolites, i.e., bacterial toxins and mycotoxins; EMAN, 2015; Martinovic et al., 2016) and can have devastating health and economic consequences in both developed and developing countries (Pires et al., 2012; EFSA, 2014; ECDC, 2015a; Henao et al., 2015). A major fraction of FBDs are diarrheal diseases, with particularly high impact on children (Pires et al., 2015). Typical FBPs are bacteria and several viruses, but also parasites and some fungi can cause FBDs.

### Conventional microbiological food analyses

**Figure 1:**
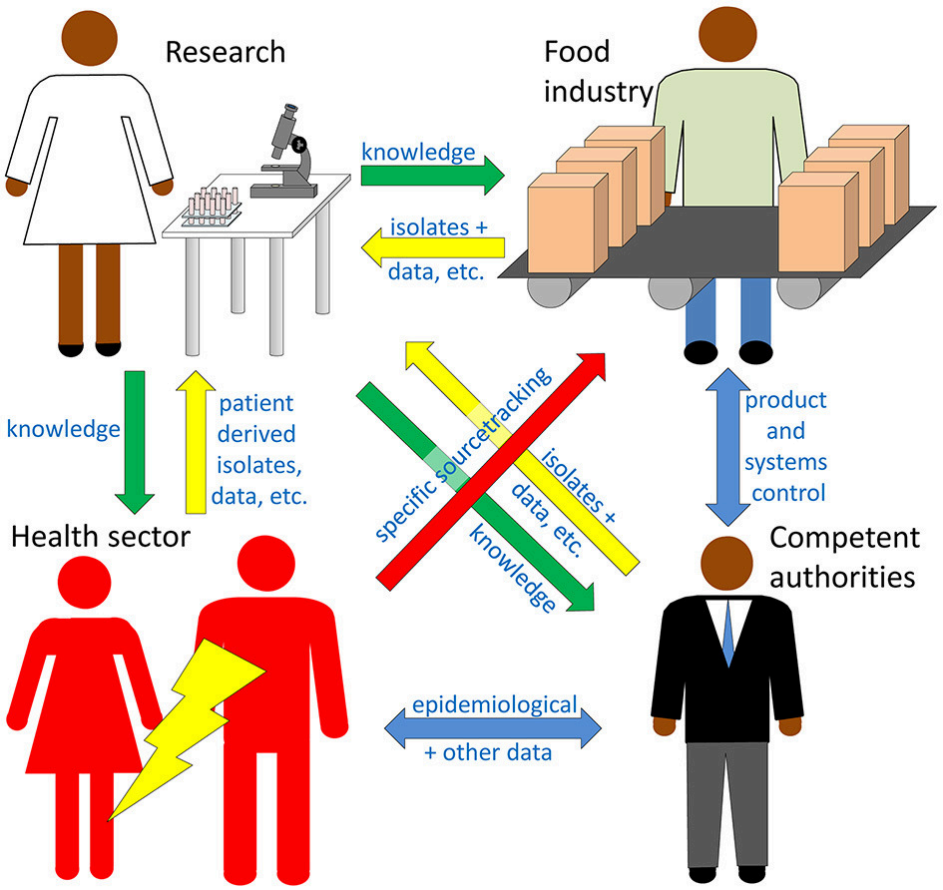
Four sectors are considered here as potential users of high throughput sequencing (HTS) technologies for detection and characterization of foodborne pathogens (FBPs). Research **(upper left)** is a knowledge driver providing exploitable reference data and detection methods among others to the other three sectors (green arrows), and receives valuable data and material back from the other sectors (yellow arrows). The food industry **(upper right)** is legally obliged to take preventive measures and to monitor its products and production systems to prevent contamination with FBPs, with economy as a main priority driver. Documentation of the systematic efforts to maintain low risk (goal = pathogen free) products must be available for inspection. The health sector **(lower left)** treats patients and is usually the first to isolate and characterize outbreak-associated strains, thereby providing key information necessary for the other sectors to investigate and minimize the impact of outbreaks. The competent authorities **(lower right)** enforce the food law and surveil the food industry and products, but also coordinate the outbreak investigations based on data provided by the other sectors. Epidemiological data, legal acts and quality control documents are the main information sources used and shared by the competent authorities (blue arrows). Outbreak investigations have a strong focus on specific source tracking (red arrow).

Microbiological analyses of foods are carried out for verification and control, surveillance, investigation of disease outbreaks or sporadic cases, or for research (Figure 1). Time and labor consuming culture dependent methods, including enrichment and/or selective steps, are often used. Selective enrichment may be crucial to capture the species of interest. Isolation of the pathogen of interest is an optimal starting point for further characterization and research, and contributes to ensure that public health agencies can perform their basic mandate of FBD surveillance and response (Forbes et al., 2017). Over the last decades traditional culture dependent methods have gradually been complemented with molecular analytical methods. Speed and costs are the main drivers of this development. Concerns about the possible lack of necessary sensitivity, specificity or correspondence between molecular findings and presence or absence of viable, pathogenic microorganisms are among factors restraining this development. Polymerase chain reaction (PCR) analysis of enriched samples from food is established for a range of FBPs improving the efficiency of screening of samples, but viable microorganisms are, in most cases, still required for definitive confirmation of positive samples. High throughput sequencing (HTS) based workflows now gradually emerge as options also for routine applications to FBP detection and characterization (Figure 1; Table 1).

**Table 1:** Users, criteria and limitations on use of analytical methods for detection of foodborne pathogens^a^.

	**Food industry, quality control**	**Public enforcement, legal/safety control**	**Clinical outbreak investigation, source tracking^b^**
Molecular targets	A narrow range of pre-defined risk indicators, e.g., particular species specific markers, serotype specific markers, virulence genes	A broader range of risk indicators, pre-defined or not. May also include focus on different sequence variants	At least all case specific markers identified from characterization of patient isolate(s). Often also including a broad range of other risk indicators, pre-defined or not
Purpose of analysis	Ensure that own food product is legally compliant and does not pose an unacceptable risk to consumer (can be perceived as safe)	Ensure that food production/products are legally compliant and safe, and monitoring of prevalence/distribution of contaminants on the market	Identify contaminated product(s) and retract the product(s) from the market as well as clearing non-contaminated products from suspicion
How?	Analytical verification: pre-defined risk indicators are not detectable at a pre-defined limit of detection or in a pre-defined sample size. Sampling based on HACCP^c^ approach	Analytical detection and typing of risk indicators, analysis of traceability documentation and epidemiological analysis. A pre-defined sample size or LOD^d^ and quantitative analyses are often applied. Sampling based on specific sampling plans, standards or guidelines	Interviews of patients/family 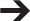 analytical detection targeting case specific markers from patient isolate(s) complemented with epidemiological analysis and sampling partly based on interviews. A pre-defined sample size is often applied, but a pre-defined LOD is usually not
Required time from test to result	Minutes or usually several hours, but may be justifiable with up to days/weeks in case of re-emerging contamination problem	Exceptionally weeks, usually days, but may be shorter in case of products with short shelf life and high turnover	As soon as possible, preferably minutes to hours, but can be days
Resource limitations	Routine on-site detection. Price sensitive market 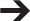 testing costs per unit produced must be very low, but may be justifiable with high costs in case of re-emerging contamination problem	Routine laboratory analyses complemented with in-depth analyses in well-equipped (reference) laboratories. Testing costs per unit can be low to moderate, depending on specific control program. High to very high costs can be justified in particular cases	Emergency conditions: life and health of humans are at stake. Routine and in-depth analyses in advanced laboratories. Testing costs per unit can be moderate to very high
Representative analytical approach	Traditional culturing, PCR^e^ or immuno-assay analyses	Enrichment culturing, biochemical tests and PCR complemented with sequencing of genes	PCR complemented with gene sequencing and WGS^f^ of selected isolates
High throughput sequencing (HTS) applicable? When?	Usually not because of costs and time. May be justifiable to apply HTS based genome typing methods on isolates to investigate/unravel re-emerging contamination problems	Usually not because of costs and time. HTS based amplicon sequencing of one to a few genes in some cases justifiable. Selected isolates from public enforcement/control programs occasionally qualify for WGS	Yes, typically by WGS of selected isolates, possibly limiting bioinformatics to focusing on particular gene panels while facilitating successive in-depth analyses of genome evolution and epidemiology

a *Research applications are essentially unlimited and therefore not included in this table*.

b *Patient treatment and characterization of patient derived isolates is a separate task not included in this table. However, the source tracking rely in part on the data derived from characterization of patient derived isolates*.

c *HACCP, hazard analysis (and) critical control point*.

d *LOD, limit of detection*.

e *PCR, polymerase chain reaction*.

f *WGS, whole genome sequencing*.

### High throughput sequencing

**Figure 2:**
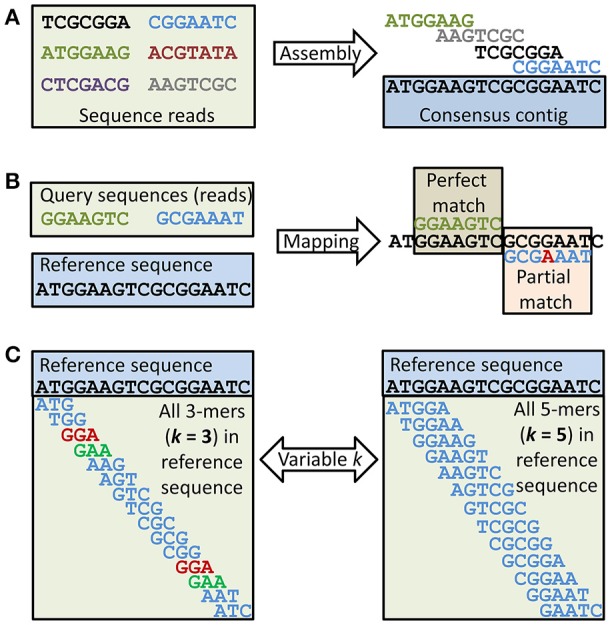
Metagenomics data analysis. At least three different approaches for analysis of HTS sequence reads can be selected, but combinations are often preferred. **(A)** Assembly of sequences (e.g., reads) into contigs (consensus sequences) requires mapping. Sets of contigs are often further assembled into scaffolds (not shown), where the relative position of contigs is known but gaps of ± known size between the contigs remain to be closed. The example shows that four of the six reads can be assembled into a consensus contig while the two remaining reads cannot be assembled with any of the others. **(B)** Mapping of sequences (e.g., reads) to other sequences (e.g., in database) also requires mapping. The example shows one perfect and one partial match between two query sequences (e.g., reads) and a reference sequence. The mismatch in the partial match is shown in red. **(C)** Any sequence larger than one nucleotide can be divided into subsequences of length *k* ≥ 1. The size of *k* will affect the likelihood of any random *k*-mer being unique to a data set. A small *k* will reduce the number of unique *k*-mers. This is demonstrated in the example, as for the given reference sequence two *k*-mers will not be unique with *k* = 3, while with *k* = 5 all *k*-mers are unique. Rare *k*-mers or *k*-mer frequencies can be used to estimate relationships between two sets of sequences (e.g., two shotgun metagenomes or a sequence isolate and a reference genome).

**Figure 3:**
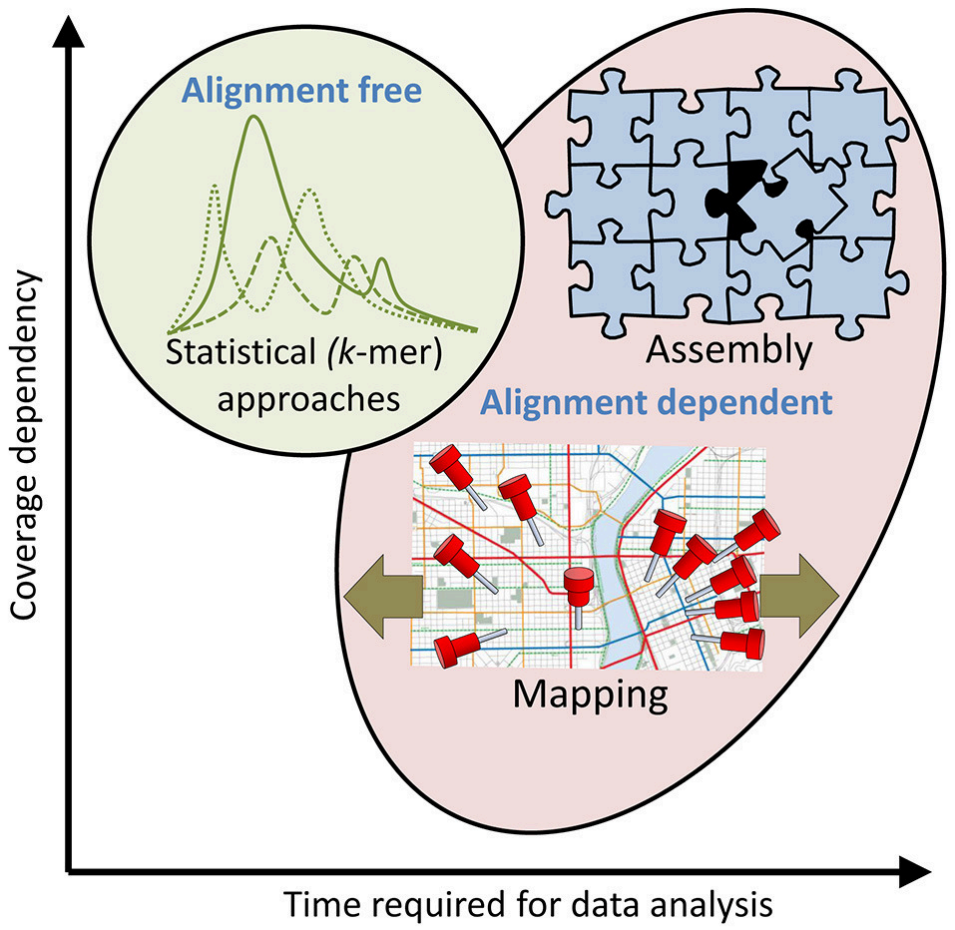
Approaches to HTS sequence read analysis and their dependence on alignment, time and coverage. At least three different approaches for analysis of HTS sequence reads can be selected, but combinations are often preferred. **Top right: Assembly** of sequences into contigs (see also Figure 2), scaffolds and complete genome assemblies is alignment dependent, time consuming and the success probability is usually correlated with the coverage. This approach is typically taken when time is not the limiting factor and a complete assembly is desired for successive analysis and reference applications. **Bottom: Mapping** (see also Figure 2) of reads to existing assembly/assemblies is also alignment dependent and time consuming but can also be performed successfully at low coverage (a single read can be mapped to a reference assembly). The size of the reference (e.g., database or genome) and the degree to which mismatches are accepted will have a significant impact on the time required for data analysis (olive arrows). This approach is typically taken to determine functional aspects of metagenome and transcriptome sequences and in metataxonomics. **Top left: *K*-mer analysis** (see also Figure 2) is a fast, alignment independent, statistical (probabilistic) approach to investigate properties of a sequenced genome such as its similarity and relationship to other (reference) genomes. It is typically used to screen sequenced genomes to identify genomes of particular interest for more comprehensive analysis.

HTS can generate thousands to millions of sequence reads, and up to several hundred billion base pairs (bp) of sequence information per sample. The read length, error rate and number of reads and sequenced bases vary substantially. Selective amplification (targeted) and non-selective, random (shotgun) approaches exist. The number of high quality genomes for the most important food pathogens is already high and rapidly growing, in part benefiting from the relatively small genome sizes of most microorganisms (≤100 Mbp). In cases where a sequenced reference genome is unavailable it is necessary to perform *de novo* sequencing and genome assembly (Figures 2, 3). *De novo* assembly to obtain a draft quality genome based on high quality, short-read (< 250 bp) sequence data from single, cultured isolates is complex, but can be (semi-)automated (Emond-Rheault et al., 2017). Closing a genome often requires highly skilled bioinformaticians and long sequence reads (up to several kbp) or PCR and Sanger sequencing of the unknown gaps in scaffolds (Goodwin et al., 2015; Loman et al., 2015; Rhoads and Au, 2015). Even this can sometimes be (semi-)automated (Emond-Rheault et al., 2017). Analysis of re-sequencing data employing a mapping strategy (Figures 2, 3) is less complex, and can be (semi-)automated. It is, however, time and computer intensive and interpretation of the mapped data can be quite challenging (Goodwin et al., 2015; Loman et al., 2015; Rhoads and Au, 2015). Alignment-independent comparisons using statistic (probabilistic) approaches (*k*-mers; Figures 2, 3) can be much faster and automated and emerge as an attractive option at the cost of detailed resolution (Ondov et al., 2016). The optimal HTS strategy is therefore purpose dependent. Large sequence databases including thousands of completely sequenced genomes are now available, facilitating identification of common as well as rare but potentially important genes by mapping of sequence reads to the database sequences. Low error rate, long reads and high coverage facilitate sequence assembly (Figures 2, 3). Altogether, this has expanded our ability to gain a more comprehensive insight into the genetics of individual strains or species, and the microbiota (microbial species composition) and microbiome (functional gene pool of microbiota) of a very broad spectrum of sample types, including environmental, food and clinical samples.

Tremendous progress in HTS technology developments has been made during the recent decade and this review will not discuss *per se* the sequencing technologies, because several excellent reviews are available (Loman and Pallen, 2015; Goodwin et al., 2016). Understanding advantages and disadvantages associated with the different HTS platforms, (in particular the read length, read number, sequencing error rate and costs) may, however, help readers to better understand the choices made in the following cited examples.

Illumina sequencing is currently the prevailing HTS technology and also offers the highest fidelity. It provides very large data sets of relatively short reads (100–300 bp) at an error rate per sequenced base of approximately 1%. Roche 454 (phased out in 2016) was the previously dominating technology and provided smaller data sets of longer reads (≤700 bp) with higher error rates. Much of the initial HTS literature reports on Roche 454 data (Liu et al., 2012; Mayo et al., 2014; van Dijk et al., 2014). Long read sequences, up to several kbp, can be obtained using other platforms (see below) but the error rates are high (5–40%). All HTS technologies allow for assembly of good quality draft bacterial genomes with up to 100 contigs. A fully closed genome sequence is usually obtained through application of different technologies combining the accuracy of short reads with the ability of long reads to span gaps in assembly scaffolds (Tallon et al., 2014). Due to the capacity of the Single Molecule Real Time sequencing technology of Pacific Biosciences to provide reads of 1–10 kbp, this technology has recently been popular for *de novo* assembly of completely closed genomes, long repetitive sequences, plasmids and bacteriophages (Rhoads and Au, 2015). The Oxford Nanopore MinION has competitive potential, as it is claimed to be able to provide even longer reads in real-time at low costs (Goodwin et al., 2015; Loman et al., 2015; Quick et al., 2015). The short read technologies generally provide significantly higher coverage than the long read technologies, at much lower costs per sequenced base.

### HTS based microbiological analyses

**Figure 4:**
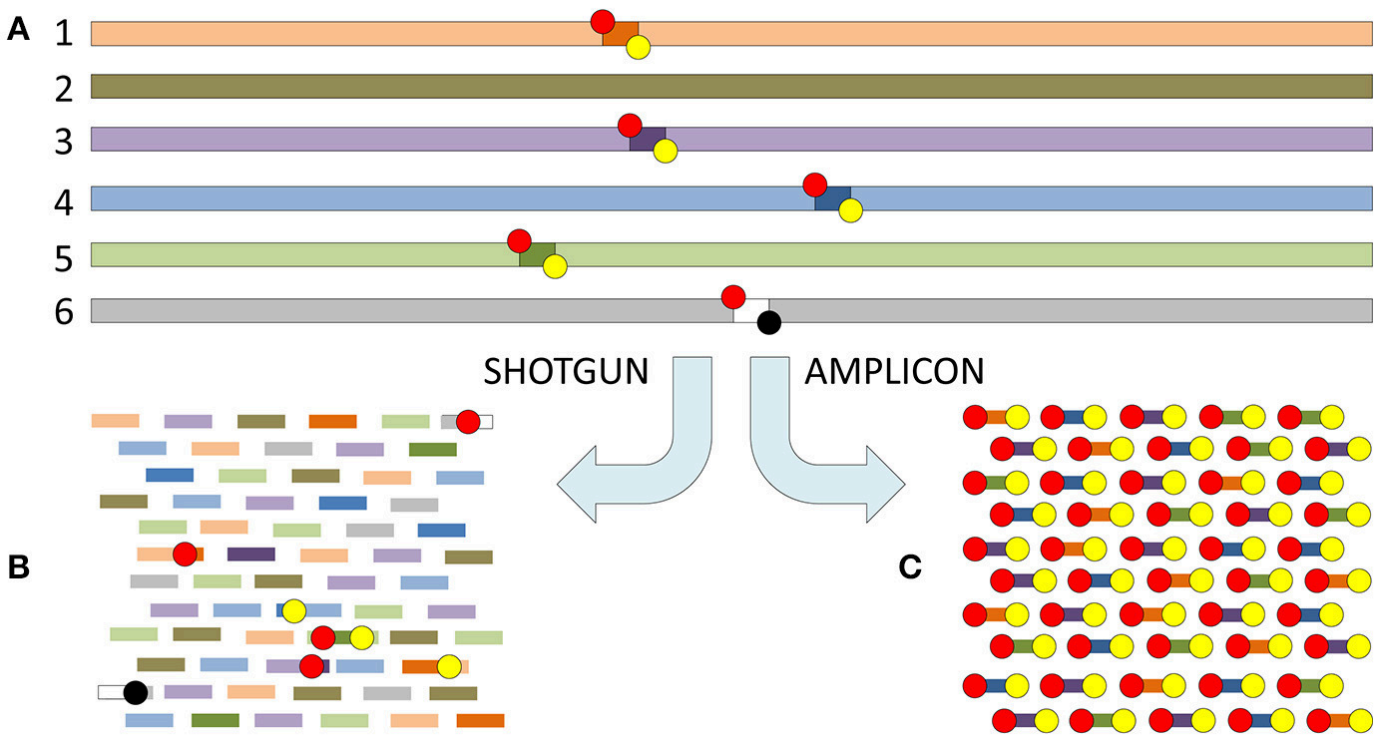
The difference between shotgun metagenomics and amplicon based metataxonomic sequencing. **(A)** Six different genomes (1–6) shown in different colors with five of the six (1 and 3–6) containing a shared genomic region. The shared genomic region in all five has a conserved motif on the left side (red circle = forward primer binding site), but one of them has a significant change in the conserved motif on the right side (yellow circle = reverse primer binding site) resulting in primer mismatch (black circle). **(B)** The sequenced fragments with shotgun metagenomics are random motifs from the six different genomes, and only one of the conserved (primer binding) motifs will exceptionally be included. **(C)** The sequenced fragments with amplicon sequencing are only those delimited both by a conserved left and right motif (red and yellow circles). The difference in mean coverage per nucleotide is significant. Assuming that each genome has the same length (e.g., 10^8^ bp) and is present in equal concentration in the original sample, a read length of 200 bp, and an invariant length of the shared genomic region delimited by the primer sites of 250 bp, the mean coverage per nucleotide of the targets will be: **(B)** *R* × *L*/*N* × *G* = 10^6^ × 200 bp/6 × 10^8^ bp = 1/3 where *R* = number of reads, *L* = read length, *N* = number of genomes and *G* = genome size. **(C)** *R* × *L*/*A* × *D* = 10^6^ × 200 bp/4 × 250 bp = 2 × 10^5^ where *R* and *L* are the same as for B, while *A* = number of genomes flanked both by conserved forward and reverse primer sites, and *D* = the length of the shared genomic region delimited by the primer sites. In this example C is 6 × 10^5^ times more sensitive than **B**. Shotgun reads may be analyzed applying all bioinformatics approaches (assembly, mapping and *k*-mer analysis, alignment dependent and alignment free; cf. Figures 2, 3). Amplicon reads are usually analyzed by mapping, clustering and phylogenetic approaches, while assembly is only exceptionally applied.

Foods, feeds, clinical and environmental samples harbor complex and diverse microbial communities. We use the expression “metagenomics” for analysis of such samples by HTS. For further distinction we use the term “shotgun metagenomics” for whole genome sequencing (WGS) or whole-sample-DNA based metagenomics (Figure 4). Targeted amplicon analysis of various ribosomal RNA coding DNA (rDNA) and other conserved markers is, as suggested by Marchesi and Ravel (2015), denoted “metataxonomics” throughout this review. “Metabarcoding” is a related term sometimes used in the literature (e.g., Jones et al., 2013; Staats et al., 2016). Shotgun metagenomics, and “metatranscriptomics” (i.e., shotgun sequencing of RNA transcripts) enables full genome or transcriptome sequencing, respectively, in a complex sample. RNA based shotgun metagenomics (i.e., sequencing of all RNA in the sample) and metatranscriptomics are often combined into a single approach “RNAseq”. We therefore only refer to metatranscriptomics or RNA based shotgun metagenomics when we need to distinguish from RNAseq.

Metataxonomics is generally more sensitive than shotgun metagenomics, due to enrichment of targets by amplification. Metataxonomics is, due to the targeted enrichment, prone to bias and may fail to detect novel variants of relevant targets, e.g., 16S rDNA with mismatches in the PCR primer binding motifs or genes involved in previously unknown but relevant biosynthetic pathways. Shotgun metagenomics on the other hand presumably has a low bias, is independent of *a priori* knowledge of target sequences, and can be used to monitor alterations in the microbiome that may not be evident from the composition of the microbiota. RNAseq is biased by the reverse transcription used to synthesize cDNA prior to sequencing, possibly affecting detectability of RNA viruses. Metatranscriptomics is further biased by RNA transcription rates. RNAseq is otherwise comparable to shotgun metagenomics. The main drawbacks of shotgun metagenomics are: several logs higher (inferior) limit of detection (LOD; Figure 4) and complex data analyses (bioinformatics) that, at present, are difficult to automate/standardize. The composition of the sample's microbiota and/or the causative agent is typically not well known in advance (limiting the possibility to apply targeted approaches such as metataxonomics), and relevant reference sequences may be lacking from public databases (limiting the possibility to perform mapping and assign function to sequences).

## Selected, illustrative examples of approaches for specific pathogen taxa and applications

FBDs are caused by bacterial, viral, fungal or parasitic pathogens entering the body via contaminated foods and have entered the food chain at some point from farm to fork. Bacteria and viruses are the most commonly reported sources of disease. Bacteria are more easily identified than viruses because the former can often be cultured. The vast majority of published HTS based FBP studies have focused on particular taxa, mainly bacterial, and specific applications, e.g., outbreak investigation starting with a clinical isolate. The organization of the following sub-chapters reflects this. Future advancements, with some included pioneer examples, are expected to allow for simultaneous detection of multiple higher and lower level taxa.

### Bacterial foodborne pathogens

Many FBPs are well-studied and the use of genomic data and large scale WGS have become important for studies in epidemiology, evolution, surveillance and outbreak investigations. On August 16th 2017, there were 104,667 (84,726) genome assemblies and 8,119 (6,286) complete bacterial genomes (number in brackets was 7 months earlier) available from National Center for Biotechnology Information (NCBI; http://www.ncbi.nlm.nih.gov/genome/browse/). The contribution of bacteriophages to bacterial genome size, evolution and virulence is very significant (Brüssow et al., 2004; Salmond and Fineran, 2015). Shotgun metagenomics can provide new insights into these aspects and the possible relevance of phages to FBDs (Nieuwenhuijse and Koopmans, 2017).

Bacterial FBPs are often present in foods in low numbers heterogeneously spread in the product. Consequently, ability to detect very low levels of FBPs in various food sources is important. Current detection methods normally involve one or more enrichment steps, screening (e.g., by PCR), followed by an isolation step. Isolation of a FBP from a food matrix may be challenging due to low recovery of isolates;—a sample can be positive with PCR screening, yet isolation of a corresponding bacterial strain may not be achieved. Some of the most important bacterial FBPs are *Salmonella, Listeria monocytogenes* and Shiga toxin-producing *E. coli* (STEC) causing many outbreaks and sporadic cases with severe or fatal outcome (Crim et al., 2014; Astridge et al., 2015; EFSA, 2015b). These three FBPs are used in the following as illustrative examples. For *Salmonella* infections contaminated food sources typically include poultry, eggs, swine and ready-to-eat foods, and affect people at all ages (EFSA, 2015b). *L. monocytogenes* is commonly detected in ready-to-eat foods such as smoked fish and soft cheeses and often affect elderly, immunocompromised patients, pregnant women and have high mortality rate (EFSA, 2015b). The main food vehicles of STEC infections are bovine meat followed by vegetables and juice (EFSA, 2013, 2015b). STEC can cause severe complications like acute kidney failure (hemolytic uremic syndrome) and often affects children under the age of five, elderly and immunocompromised people (Davis et al., 2014).

For outbreak investigation the pathogen must be linked to the correct food product (source of infection). Food producers on the other hand, need to determine if their products or production line is contaminated, how an unwanted pathogen entered their production facilities, and/or if it is a persistent household strain (Figure 1; Table 1). Several strategies can be applied for comparison of isolates, e.g., pulsed-field gel electrophoresis (PFGE), multi-locus variable number of tandem repeats analysis (MLVA) and multi-locus sequence typing (MLST). For many FBPs the traditional typing or subtyping offers too low (phylogenetic) resolution to distinguish closely related but distinct strains. High resolution is required to discriminate parallel outbreaks or to separate sporadic cases from an outbreak, but also to assess if a reemerging contamination problem is caused by a persistent strain or reintroduction of similar strains.

WGS will provide highly discriminatory data for subtyping of strains by single nucleotide polymorphism (SNP) analysis or extended (core genome or whole genome) MLST (cgMLST, wgMLST) for strain comparison for outbreak investigations and surveillance purposes. Among possibilities beyond traditional molecular fingerprinting is the reanalysis of complete genome sequences when subsets (e.g., MLST) provide insufficient information/resolution. Polymorphisms can be investigated with or without mapping of the HTS data to a reference genome (Figures 2, 3). WGS-based analyses can also aid in the identification of other relevant factors such as virulence and antibiotic resistance genes (Joensen et al., 2014; Holmes et al., 2015; Octavia et al., 2015; Forbes et al., 2017). By standardizing the workflow of the actual HTS and bioinformatics analysis, this can take only a few days. However, comparable data and standardized protocols and pipelines are required, a topic discussed in further detail below.

### Bacterial genomic (isolate and strain typing) approaches

#### Outbreak investigations

The starting points for outbreak investigations with strain typing are access to clinical isolates. WGS has been used many times in recent years for comparison of isolates in outbreak investigations. Most published studies were retrospective, but a few were performed in real-time. A selection of examples is summarized in Table 2. In an early prospective study (2009) isolates from human patients and animals associated with an STEC O157:H7 outbreak were selected for WGS for comparison of isolates and source identification (Underwood et al., 2013; Table 2). A combination of Roche 454 and Illumina data were used to generate a reference assembly from the strain with best quality data. The hybrid assembly resulted in 463 contigs with average size of 12,028 bp and served as a reference genome for the successive analysis. Shotgun metagenome reads from 16 isolates associated with the outbreak were mapped and examined for SNPs over the entire genome. Based on the SNP results five subtypes of the outbreak strain were identified, providing for design of assays for detection of six specific SNPs. These assays were used to follow the outbreak, including analysis of 106 additional isolates obtained from the outbreak, demonstrating that the five subtypes were widely distributed on the involved farm prior to the first human clinical case (Underwood et al., 2013). Variable number of tandem repeats and PFGE typing indicated that there were two different strains in the sample collection, but the data from each typing method did not overlap and were therefore inconclusive. The HTS data on the other hand documented that the outbreak was caused by a strain differing in four SNPs from the hypervirulent O157:H7 ST11 clade 8. This early study demonstrated that HTS can provide better resolution (five subtypes vs. two subtypes) and therefore can be superior to more traditional characterization methods. HTS in addition provided data suitable for design of specific diagnostic assays that improved the monitoring of the outbreak.

**Table 2:** Examples^a^ of published high throughput sequencing based investigations of foodborne pathogen (FBP) outbreaks.

**Type of FBP**	**Isolates/strains or community analyzed?**	**Material sequenced**	**Sequencing technology applied**	**Sequencing and bioinformatics approaches**	**Where and when**	**References**
**BACTERIAL FBPs**						
*E. coli* (STEC^b^) serotype O157:H7	Isolates	70 isolates from two outbreaks complemented with isolates from sporadic cases	HiSeq2500 (Illumina), 200PE^c^	Whole genome sequencing (WGS) of single isolates followed by mapping to reference genome and single nucleotide polymorphism (SNP) analysis	UK, 2013	Jenkins et al., 2015
O157:H7	Isolates	29 isolates, 24 isolates from an outbreak, 5 unrelated cases	MiSeq (Illumina), 150PE	WGS of single isolates followed by *de novo* assembly and reference-based SNP analysis	US, 2001–2012	Turabelidze et al., 2013
O157:H7	Isolates	16 isolates, 8 from humans, 8 from animals	GS FLX (Roche) and GAIIx (Illumina)	WGS of single isolates followed by *de novo* assembly and reference-based SNP analysis	UK, 2009	Underwood et al., 2013
O157:H7	Isolates	105 isolates, 10 human isolates from an outbreak, 95 human isolates from sporadic cases	Ion Torrent PGM (Life technologies)	WGS of single isolates followed by mapping to reference genome and single nucleotide polymorphism (SNP) analysis	UK, 2007–2012	Holmes et al., 2015
Multiple STEC serotypes	Isolates	46 isolates, 6 isolates from humans from an outbreak, 40 human isolates from sporadic cases	Ion Torrent PGM	WGS of single isolates followed by multi-locus sequence typing (MLST), *k*-mer and phylogenetic analysis against up to 5,029 bacterial genomes	DK, 2012	Joensen et al., 2014
O104:H4	Communities	45 clinical human fecal samples	MiSeq 151PE and HiSeq 2500 151PE rapid mode shotgun metagenomics yielding from 8.6 × 10^6^ to 4.4 × 10^7^ reads per sample	Retrospective study applying shotgun metagenomics, followed by *in silico* subtraction of human DNA and *de novo* assembly to create environmental gene tags (EGTs). EGTs identified in more than 20 fecal samples were selected for further analysis, and EGTs identified in fecal samples from healthy humans were discarded. Reference-based mapping and phylogenetic analysis combined with further assembly to functionally annotate the EGTs and reconstruct the outbreak strain genome. Verification of results by comparison to the previously sequenced genome of the outbreak strain	Germany/Europe, 2011	Loman et al., 2013
*Listeria monocytogenes*	Isolates	2 isolates with one band difference on PFGE	GS FLX and GAIIx	WGS of single isolates followed by *de novo* assembly and gap closure	Canada, 2008	Gilmour et al., 2010
*L. monocytogenes*	Isolates	5 isolates, three from humans and two environmental isolates associated to one outbreak	Ion Torrent PGM	WGS of single isolates followed by *de novo* assembly and scaffolding against an assembled reference genome	Australia, 2013	Wang et al., 2015b
*L. monocytogenes*	Isolates	18 isolates, 7 human, 10 food isolates, 1 control isolate	MiSeq, 75PE, 150PE and 250PE	WGS of single isolates followed by *de novo* assembly and gene-by-gene analysis (extended MLST)	Austria 2011–2013	Schmid et al., 2014
*Salmonella enterica* serovar Typhimurium	Isolates	57 isolates from 5 outbreaks	MiSeq, 250PE	WGS of single isolates followed by *de novo* assembly and mapping based SNP analysis	Australia, 2006–2012	Octavia et al., 2015
Serovar Typhimurium	Isolates	61 isolates, 21 human and 5 food isolates related to an outbreak and additional 35 isolates	HiSeq2500	WGS of single isolates followed by mapping based SNP analysis	UK, 2012	Ashton et al., 2015
Serovar Enteritidis	Isolates	55 isolates, 28 isolates from 7 outbreaks, 27 isolates from sporadic cases	MiSeq, 250PE	WGS of single isolates followed by mapping based SNP analysis	US, 2001–2012	Taylor et al., 2015
Serovar Enteritidis	Isolates	6 isolates, 3 human and 3 food isolates	HiSeq2000 (Illumina), 100PE	WGS of single isolates followed by *de novo* assembly and mapping based SNP analysis	Belgium, 2014	Wuyts et al., 2015
Serovars Typhimurium, Enteritidis and Derby	Isolates	47 isolates, 26 isolates from 9 outbreaks, 21 isolates from sporadic cases	GAIIx, 101PE	WGS of single isolates followed by reference free *de novo* assembly, complemented with reference based pan-genome, SNP and *k*-mer analysis	Denmark, 2000–2010	Leekitcharoenphon et al., 2014
**VIRAL FBPs**						
Hepatitis A virus (HAV)	Isolated HAV communities	HAV communities from clinical serum and fecal samples from 120 patients associated with a single outbreak	No data on sequencing approach except fragment size (315 bp)	Metataxonomics, i.e., amplicon sequencing of VP1/P2B region of HAV, followed by mapping to HAV genotype reference sequences, complemented with phylogenetic analysis	USA, 2013	Collier et al., 2014
HAV	Isolates and communities	Two food samples from two apparently unrelated HAV outbreaks	RNAseq combined with amplicon sequencing (MiSeq; 250PE)	Four amplicons, partially overlapping, covered the entire HAV genome. Genome assembly followed by genotyping by mapping to HAV reference genomes	Italy, 2013	Chiapponi et al., 2014
Rotavirus	Community	One clinical fecal sample from a small outbreak	RNAseq on MiSeq (120PE), approximately 1 × 10^6^ reads	Genotyping by mapping to rotavirus reference genomes	Japan, 2012	Mizukoshi et al., 2014
**FUNGAL FBPs**						
*Mucor circinelloides* f. *circinelloides*	Isolates	Seven isolates from outbreak associated food (yogurt)	WGS (HiSeq2000 100PE and 100MP^d^) and amplicon sequencing (targets specified but no details on sequencing approach)	Metataxonomic MLST approach to species and sub-species (*formae*) identification, targeting three loci (ribosomal internal transcribed spacer and partial large subunit RNA gene, and RNA polymerase subunit gene), complemented with phylogenetic analysis. Whole genome comparison by genome assembly and mapping to genome sequences of two other *M. circinelloides* isolates	USA, 2013	Lee et al., 2014
**PARASITIC FBPs**						
*Kudoa septempunctata*	Community	Frozen filet of outbreak associated food (fish), and vomit samples from patients associated with the outbreak	RNAseq on GAII (80PE) complemented with Sanger amplicon sequencing of DNA (1.1 kbp fragment of 18S rRNA gene)	*In silico* subtraction of reads from the food sample against fish genome followed by mapping of remaining reads for taxonomic classification. Metataxonomic analysis of 18S rRNA gene sequence by mapping to multi-species 18S rRNA gene database	Japan, 2008–2010	Kawai et al., 2012

a *The examples are further described and discussed in the main text of this review*.

b *STEC, shiga-toxin producing E. coli*.

c *200PE refers to the use of paired-end sequencing with a read length of 200 bp*.

d 100MP refers to the use of mate-pair sequencing with a read length of 100 bp

Specific diagnostic sequence motifs are not always available or known, and a specific pathogenic agent may exhibit new and unexpected combinations of involved virulence genes for which current tests are not optimally designed. This was for example the case in the large STEC O104:H4 outbreak in Germany and other European countries in 2011 (Scheutz et al., 2011). In mid-May the public health authority in Germany was informed about a cluster of three cases with hemolytic uremic syndrome and raw WGS data from a patient derived isolate were already on June the 2nd published by Beijing Genome Institute (NCBI accession no. SRX067313; Kupferschmidt, 2011). Public release of these data incited a huge joint effort from bioinformaticians and researchers around the world, very quickly resulting in in-depth knowledge of the strain. This also facilitated design of specific diagnostic tools for further investigation of the outbreak (Struelens et al., 2011).

A complex outbreak investigation in the UK identified watercress as the source of STEC O157 in two simultaneous outbreaks with different sources of contamination (Jenkins et al., 2015; Table 2). SNP positions of high quality in all genomes of the Public Health England STEC O157 database were then extracted. Pseudo sequences of polymorphic positions were used to create maximum-likelihood trees and compared to the WGS data of additional strains held in the database. Phylogenetic analysis supported a foreign source for the outbreak, but no microbiological link to a specific country of origin was identified. Only one isolate was identified from the irrigation water from the implicated watercress, indicating a low level of contamination. This isolate was compared to the human isolates, and a maximum of 3 SNP differences were reported for the second outbreak, confirming the source of this outbreak (Jenkins et al., 2015).

One of the first published studies using WGS in outbreak investigations concerned a large *L. monocytogenes* outbreak in Canada in 2008 (Gilmour et al., 2010; Table 2). Two clinical isolates with similar but distinct PFGE patterns were subjected to WGS to assess the genetic diversity of these isolates. Altogether 28 SNPs and three indels, including a 33-kbp motif corresponding to presence/absence of a prophage were observed. The additional information obtained with WGS compared to PFGE indicated that not one, but three distinct, yet closely related strains were possibly involved in the outbreak.

Investigations of an Australian hospital outbreak of *L. monocytogenes* in 2013 identified a chocolate profiterole from a specific food manufacturer as the common food consumed by the patients. A follow-up WGS study identified more SNP differences in the environmental isolates from the food manufacturing facility than the patients' isolates from the outbreak (Wang et al., 2015b; Table 2). However, the five outbreak isolates shared multiple distinctive genetic features including five prophage insertions. Wang et al. (2015b) suggested that the human isolates were less divergent because of successful adaptation to the relatively stable human environment while the environmental strains (19–20 SNP differences from human isolates) were under increased survival pressure due to less favorable conditions.

Schmid et al. (2014; Table 2) investigated a cluster of listeriosis in Austria and Germany by WGS where the human isolates shared PFGE and fluorescent amplified fragment length polymorphism profiles. Gene-by-gene comparison or cgMLST based on 2,298 genes revealed that four of the human isolates belonged to a single cluster differing by ≤6 alleles (genes). This cluster was distinct from but related to food isolates from two Austrian producers (differing by ≤8 and ≤19 alleles, respectively). The study did not explain if the allelic differences corresponded to SNPs or were more substantial. The other three human isolates were more distinct and unrelated to the outbreak cluster.

**Figure 5:**
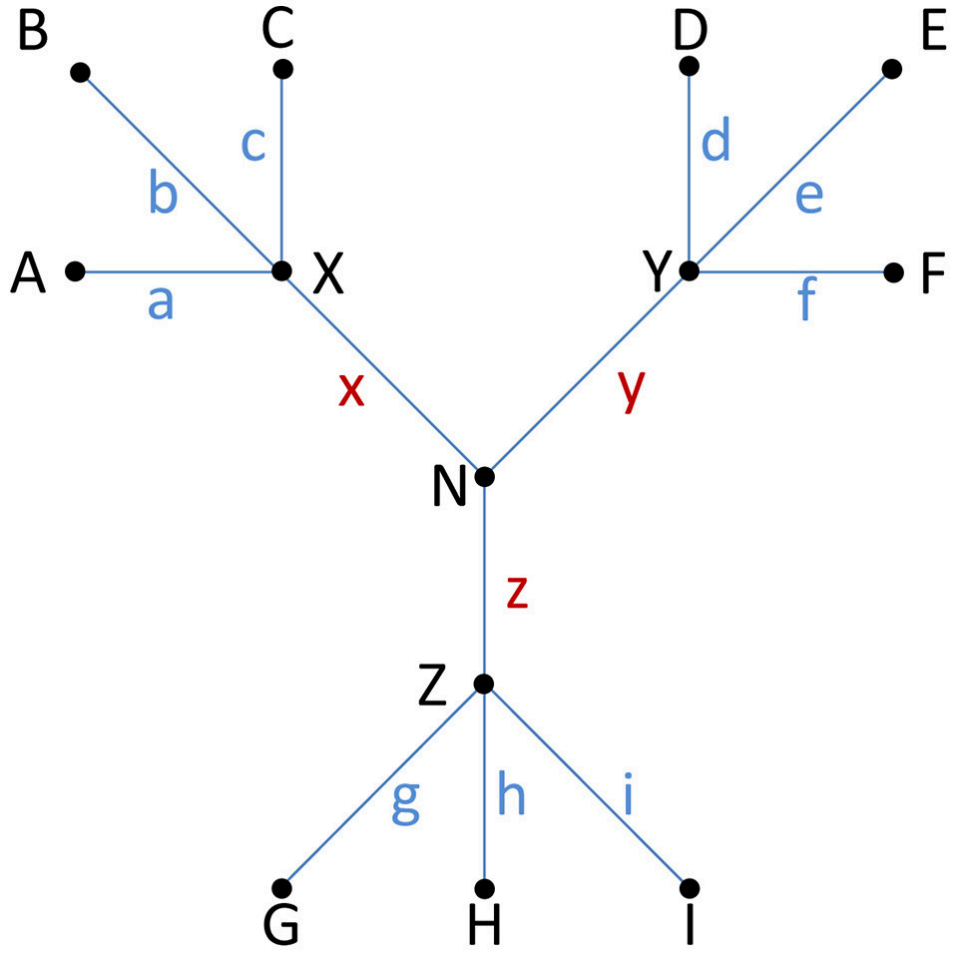
Isogenic or non-isogenic isolates? The distance or number of observed differences between isolates, usually measured as single nucleotide polymorphisms (SNPs) in HTS studies, can provide clues to determine if isolates belong to the same strain, i.e., whether they are isogenic or not. This is important for outbreak investigations, epidemiology and to assess if a persistent strain is present in a food production system. Fewer than ten SNPs is often interpreted as evidence of an isogenic origin of bacterial isolates (see examples and discussion in the main text of this paper). Practice is currently not harmonized and also depends on the taxon in question, how SNPs or other differences are calculated, and which part of the genome the study covers (e.g., core or whole genome). An inferred phylogenetic relationship between nine isolates (A–I; terminal nodes) is shown. For each isolate, a blue letter (*a–i*) indicates the number of unique SNPs associated with each individual isolate. Internal nodes labeled X–Z connect three clusters of isolates, while internode N connects all isolates. Brown letters (*x–z*) indicate the number of shared SNPs separating each individual cluster of isolates from the others. The distance (Δ) between any pair of isolates is the sum of SNPs (i.e., blue and brown letters) separating them, e.g., if *a* = 3, *d* = 2, *x* = 2 and *y* = 4 then Δ_AD_ = 3 + 2 + 2 + 4 = 11. The following two examples serve to illustrate the difference between putatively isogenic and non-isogenic clusters of isolates (with a threshold of 9 for isogenics): If *a* = *b* = *c* = *d* = *e* = *f* = 2, *g* = *h* = *i* = 3, *x* = 2, and *y* = *z* = 3 then all the isolates A–F might be considered isogenic (internal distance between any pair of isolates Δ_max_ ≤ 9), as might G-I (Δ_max_ = 6), whereas A–F might not be considered isogenic with G–I (internal distance between any members from two different clusters Δ_min_ ≥10). Similarly, if *a* = *b* = *c* = *d* = *e* = *f* = 6, *g* = *h* = *i* = 1, *x* = 1, and *y* = *z* = 3 then only isolates G–I (Δ_max_ = 2) might be considered isogenic (any other pair of isolates would yield Δ_min_ ≥11).

Octavia et al. (2015) used SNP analysis in an attempt to define whether an isolate was part of an outbreak or not and identify whether one or more strains were implicated in an outbreak (Figure 5). They modeled the mutation rate in *S. typhimurium* using 250 bp paired-end reads and estimated a cutoff value for the intra-strain number of SNPs the bacteria could have. When using a high or low substitution rate, and including a time limit of an outbreak from less than a month to up to 3 months the number of SNPs was estimated to differ from 2 to 9. Other studies have identified variable numbers of SNPs in *Salmonella* outbreaks. Several studies have reported 0–3 SNP differences in one outbreak (Ashton et al., 2015; Taylor et al., 2015; Wuyts et al., 2015). However, some outbreaks have reported to have larger SNP variation based on the core genome (Leekitcharoenphon et al., 2014). In concordance with many of the *Salmonella* reports, a low intra-strain number of SNPs (0–7) have been reported from epidemiologically linked cases of STEC O157:H7 (Turabelidze et al., 2013; Underwood et al., 2013; Joensen et al., 2014; Holmes et al., 2015; Jenkins et al., 2015; Figure 5).

#### Applications of strain and isolate typing to surveillance and control

Surveillance of specific FBPs has been ongoing in public health laboratories for a long time, and can benefit from access to clinical and/or food derived isolates. A few countries and laboratories have implemented WGS as a routine typing tool for public health surveillance (i.e., on clinical isolates) for selected FBPs (Joensen et al., 2014; Ashton et al., 2016; Chattaway et al., 2016; Lindsey et al., 2016). Implementation of WGS as a standard typing tool for isolates from foods as well, is still in a start-up phase and routinely done in very few countries.

Denmark has implemented WGS typing of *L. monocytogenes* isolates from patients and the food surveillance program. Two unexpected genetic clusters, as classified by MLST type, were identified through the WGS analysis during 2013–2015 and further analyzed for SNP differences by mapping to a reference genome of the same MLST sequence type (Lassen et al., 2016). Another study on *L. monocytogenes* has developed a gene-by-gene (cgMLST) method based on 1,748 loci among 957 genomes (Moura et al., 2016). High robustness was shown as different DNA extraction methods, library preparations and sequencing instruments were used as well as assembly-free and *de novo* assembly-based methods to ensure that the allelic profiles generated were the same despite differences in the WGS methodology.

WGS and alignment-free SNP analysis were used to differentiate between persistent and repeatedly reintroduced strains of *L. monocytogenes* in a longitudinal study of food-associated environments (Stasiewicz et al., 2015). The PFGE patterns suggested reintroduction due to observed differences. Patterns unique to single retailers or single states supported persistence or clonal spread. However, the WGS analysis revealed that the observed PFGE differences were caused by a single mobile element, suggesting persistent contamination. Identifying clonal isolates from different food-associated environments emphasize the importance of strong epidemiological data in traceback of foodborne outbreaks.

Both SNP-based approaches and cgMLST yield a high discriminatory power and are reproducible for comparison of isolates. A prerequisite for detailed typing methods is a clear understanding of what makes two bacteria isogenic (belong to the same strain or clonal lineage; Figure 5). Lack of harmonization complicates the conclusive linking of clinical and food isolates, epidemiology and tracing and tracking of contamination in food processing facilities. Expert opinions will depend on the bacterial species and how SNPs are calculated (whole, core or extended genome). A prerequisite when performing reference-based SNP analysis is the availability of good quality reference genomes from strains closely related to the target strain(s). Unfortunately, in case of outbreaks due to rare variants of the causative agent, such reference genomes are not always available. This is true even for some variants of pathogenic *E. coli, Salmonella* spp. and *L. monocytogenes*.

### Metagenomics for typing of bacterial communities and FBPs

Isolates are often unavailable and may be difficult to obtain. Most foods harbor complex and composite microbial communities. Contaminating pathogens are often heterogeneously dispersed and represent a minority of the microorganisms present in the sometimes complex food sample. Metagenomics approaches offer the opportunity to investigate the composition of microbes in food matrices *in toto* without selective isolation, including the detection of non-viable and “viable but not cultivable” microbes (Bergholz et al., 2014), and will capture a broader range of the microbial community than classical microbiology. Most of the numerous HTS metagenomics studies report on 16S rDNA analysis, i.e., what we refer to as metataxonomics. This approach has proven useful for identification of bacteria, phylogenetic studies and characterization of bacterial communities in different foods, water and other environments (Mayo et al., 2014; Kergourlay et al., 2015; Tan et al., 2015). However, the 16S rDNA has limited resolution power and cannot be used to detect non-bacterial taxa. Reliable 16S rDNA based classification of bacteria rarely extends beyond phylum, group or genus level (Livezey et al., 2013). A few FBPs can be identified by 16S rDNA sequencing, but only exceptionally at the species level and never to pathotype. All *Salmonella* species are considered as pathogens (Jarvis et al., 2015; Zhang et al., 2015), but only two of the *Listeria* species known so far are pathogenic, i.e., *L. monocytogenes* and *L. ivanovii*. These species are partly distinguishable based on 16S rDNA sequence. In contrast, 16S rDNA sequences cannot distinguish STEC from non-diarrheagenic or commensal *E. coli*. For identification of STEC, detection of virulence-associated genes including the Shiga toxin- and intimin-encoding *stx* and *eae* genes will be essential for meaningful strain typing. Database limitations (not all relevant taxa and haplotypes represented + possible erroneous sequences and taxonomic annotations) and the common use of only a subsection of the 16S rDNA (missing or covering only some of the inter-strain variation) further reduces the fitness of metataxonomics for FBP detection (Adeolu et al., 2016; Singer et al., 2016).

Shotgun metagenomics of the entire DNA present in a sample offers a more comprehensive insight into the microbial diversity of a sample with regard to the richness of microbial taxa at all levels, or with regard to the presence of gene families or biomarkers in general (Ferri et al., 2015; Blagden et al., 2016; Ranjan et al., 2016). Shotgun metagenomic approaches used for improved FBP detection have been tested in a few studies. Bioinformatics methods to distinguish two genomes of the same species in a complex sample are needed, but it seems possible to determine whether one or more FBP strains is involved (Leonard et al., 2016). In investigations where it is essential to detect a specific organism assumed present in low numbers in a complex matrix, culture-based bacterial enrichment is still necessary. Then, inevitably, an enrichment bias of the composition of the bacterial community relative to the original sample will emerge.

#### Shotgun metagenomics in outbreak investigations

**Figure 6:**
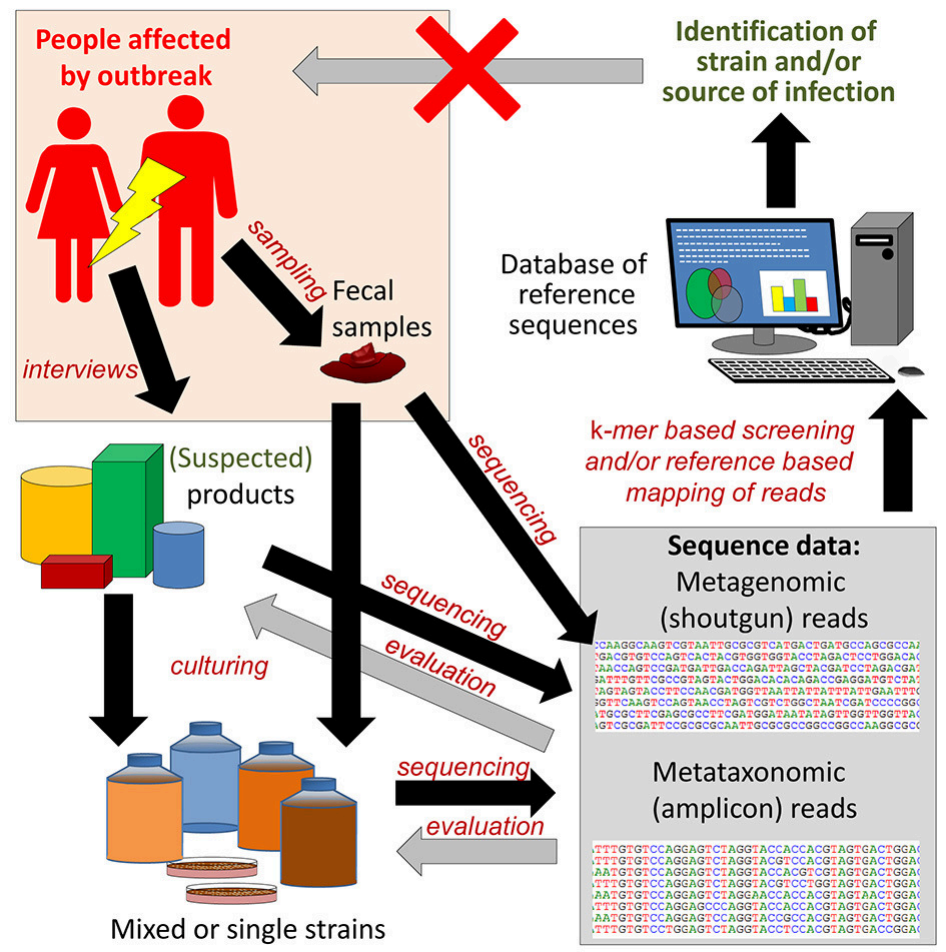
Reference guided metagenome sequencing based approach for identification and characterization of pathogenic and outbreak associated strain(s). In case of an outbreak, fecal samples from patients are subjected to culturing, in order to isolate the outbreak strain. Patients are also interviewed in order to try to identify food products that may be the source(s) of infection. The metagenomes of stool samples, food products and cultured strains can then be amplicon sequenced (metataxonomics) or shotgun sequenced (metagenomics) and the data mapped to reference databases for identification of virulence markers. Shotgun reads can also be assembled into larger contigs or genomes for identification of pathogenic strains. The latter is facilitated if the sequence data are derived from single isolates. Black arrows indicate forward flow direction of the analysis, while gray arrows indicate feedback changing the premises for earlier steps. Feedback from the sequencing analysis can be used to refine and narrow the search for a specific FBP. If successful, the outbreak will be terminated. This review includes multiple examples of the application of the described approach to outbreak investigations.

**Figure 7:**
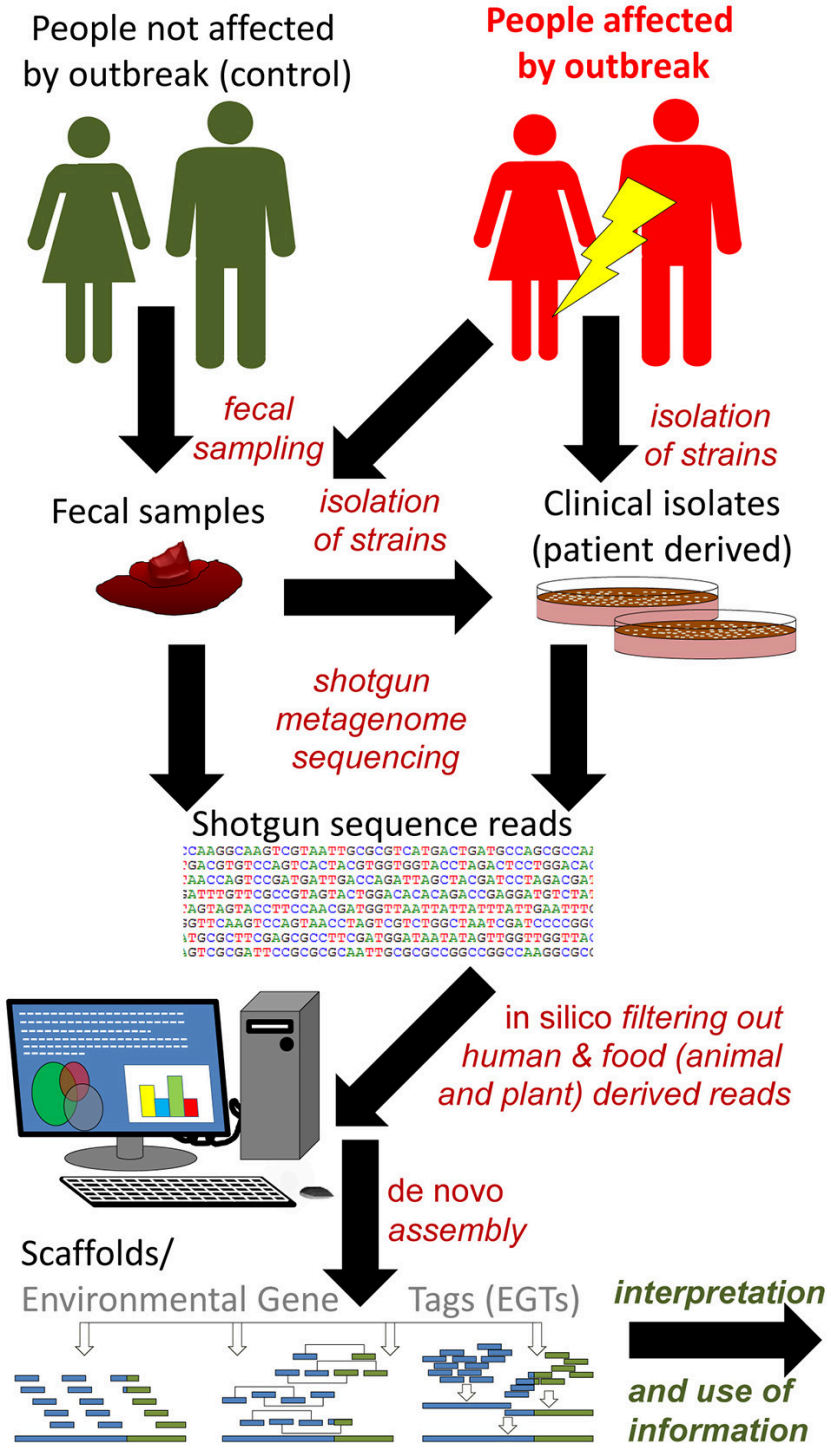
Reference independent shotgun metagenome sequencing based approach for identification and characterization of outbreak strain(s). In case of smaller outbreaks the possibility to compare metagenomes from affected people (patients) and healthy controls is limited. In these cases the availability of clinical isolates may be required to avoid exhaustive open ended bioinformatics (*in silico*) analyses, as exemplified by Brzuszkiewicz et al. (2011) and Rasko et al. (2011). Environmental gene tags (EGTs) from metagenomes of people affected by the outbreak and controls (people not affected) can be compared in case of a larger outbreak, as exemplified by Loman et al. (2013). In that study, EGTs present only in affected patients were characteristic of the outbreak strain and provided sufficient information to near complete characterization of its genome. Scaffolds and in particular assembled genomes may and should be uploaded to reference database(s), for successive use in analytical approaches like those described in Figure 6.

When screening of food products and even in outbreak investigations, the genome sequence of the specific FBP strain/strains is usually not available. HTS technology may be used to identify the genome sequence of the causative agent of the outbreak by *de novo* assembly of sequence reads from complex samples with high prevalence of the agent, e.g., clinical specimens or food samples (Figures 6, 7). Theoretically, the approach shown in Figure 6 can also be applied to control of food products by the manufacturers or enforcement authorities. However, the current costs and other resource requirements (skills, lack of standardized data analyses, time) prevent justification of the approach for routine controls.

In a retrospective study of the German/European STEC O104:H4 outbreak in 2011 it was demonstrated that such an approach could be used to identify the infectious agent in human fecal samples (Loman et al., 2013; Table 2). Forty-five fecal samples from patients were sequenced by shotgun metagenomics. Human DNA was subtracted *in silico* and assembly was performed to create environmental gene tags (EGTs). EGTs found in more than 20 of the fecal samples were selected for further analysis. The total outbreak metagenome was screened for sequence reads from healthy humans and matching EGTs were subtracted. A set of 450 outbreak-specific EGTs were then subjected to taxonomic analysis and almost 65% were assigned to the Enterobacteriales. The original metagenomics data sets were then used in an attempt to reconstruct the *E. coli* outbreak strain genome. Functional annotation confirmed the presence of important strain-specific and virulence-associated genes, and ten samples had more than 10 × coverage of reads mapped to the reference genome of the specific outbreak strain. The coverage was > 1 in 26 samples and Shiga toxin genes were detected in 27 of 40 STEC-positive samples. In some of the individual samples sequences from other human pathogens such as *Campylobacter, Salmonella*, and *Clostridium difficile* were also identified. This study indicates the potential of shotgun metagenomic analyses for the culture-independent identification of bacterial pathogens in samples with complex microbial composition.

#### Shotgun metagenomics for food surveillance and control

A selection of studies is described below and details are presented in Table 3. Tomatoes have been implicated in *Salmonella* outbreaks several times, but isolation of *Salmonella* from tomatoes has only been successful a few times. Ottesen et al. (2013) used shotgun metagenomics to describe taxa associated with pre-enrichment and throughout the enrichment steps of a protocol for *Salmonella* detection in environmental tomato samples (Table 3). DNA was extracted prior to enrichment and the remaining tomato samples were enriched overnight in a universal pre-enrichment broth and aliquots successively added to two different growth media. The sequencing depth was insufficient to capture the majority of the diversity within the samples. To achieve about 1 × coverage of all genomes Ottesen et al. (2013) estimated that they would have needed approximately 250 × more sequence data. Variation among samples suggested differences in the microbial community in the starting material of the samples. An important biological finding was the significant enrichment of *Paenibacillus* sp. from uncultured to cultured samples. This taxon is known to inhibit and kill *Salmonella*. The study also identified a number of sequences as *Salmonella*-specific despite negative PCR and culture results when those samples were tested for *Salmonella*. A comparison of results from the two different applied assembly approaches showed that increased read length, contrary to what might be expected, reduced the ability to assign taxonomy. Others have made similar observations (Luo et al., 2012). It is not clear if this phenomenon is associated with database limitations.

**Table 3:** Examples^a^ of published high throughput sequencing based approaches to detection of foodborne pathogens for industrial and control purposes.

**Target organisms**	**Type of matrix and material sequenced**	**Aim**	**Sequencing approach and technology**	**Data output**	**Bioinformatics approach to data analysis**	**References**
**BACTERIA**						
*Salmonella* and microbial community	Naturally contaminated tomatoes (4 samples, 4 conditions)	Pathogen detection (*Salmonella*) and characterization of the microbial community after pre-enrichment and enrichment	Shotgun metagenomics, MiSeq (Illumina) 151PE^b^	Raw data not described. After assembly: 1.5 × 10^7^ sequences of variable length (mean = 210 bp), comprising a total of 2.6 Gbp	Taxonomic classification by reference-based analysis in MG-RAST. Pathogen detection by reference-based mapping to a database of *Salmonella* genomes	Ottesen et al., 2013
*Salmonella* and microbial community	Naturally contaminated cilantro (coriander leaves). 91 samples included in metataxonomic study, 12 samples in shotgun metagenome study	Pathogen detection and quantification (*Salmonella*) and characterization of the microbial community before and after enrichment	Metataxonomics by 16S rDNA amplicon sequencing on GS FLX (Roche) and shotgun metagenome sequencing on MiSeq (75PE) with 1 to 6 samples per run	GS FLX raw data not described. Total number of shotgun reads ranged from 6.8 × 10^5^ to 5.5 × 10^6^ per sample depending on number of samples sequenced in one run	Taxonomic classification by reference-based analysis using the RDP classifier. Pathogen detection by reference-based mapping to genome sequences of cultured isolates of *Salmonella* from the same study	Jarvis et al., 2015
*Listeria monocytogenes* and microbial community	Naturally contaminated ice cream. 3 enrichments with four replicates and 13 time points included in metataxonomic study, 6 samples included in shotgun metagenome study	Pathogen detection (*Listeria monocytogenes*) and characterization of the microbial community before, during and after enrichment	Metataxonomics based on 16S rDNA amplicon sequencing on MiSeq (300PE), and shotgun metagenome sequencing on MiSeq (PE151)	Metataxonomic data not described. Total number of shotgun reads ranged from 1.5 × 10^7^ to 8.2 × 10^7^ per sample	Taxonomic classification by reference-based analysis using Resphera Insight and subtyping of *L. monocytogenes* 16S rDNA fragments by mapping in PYNAST. Pathogen detection and shotgun metagenome analyses with Genius bioinformatics software for identification to species, subspecies and strain level by reference-based mapping to *L. monocytogenes* genomes	Ottesen et al., 2016
STEC^c^ and microbial community	Bagged spinach: spiked and unspiked samples, altogether 16 samples	Pathogen detection and quantification of STEC^c^, virulence gene detection and characterization of the microbial community before, during and after enrichment	Shotgun metagenome sequencing on MiSeq (250PE)	Total number of shotgun reads ranged from 8.0 × 10^6^ to 1.5 × 10^7^ per sample	Pathogen detection by reference-based mapping to virulence and serotype genes and STEC genomes. Microbial community analysis by *k*-mer analyses against bacterial genomes for identification of bacterial species, phylogroup or serovar	Leonard et al., 2015
STEC and microbial community	Bagged spinach: 36 spiked samples (low level)	Pathogen detection and quantification (STEC), virulence gene detection and characterize the microbial community before, during and after enrichment	Shotgun metagenome sequencing on MiSeq (250PE)	Total number of shotgun reads ranged from 1.2 × 10^6^ to 5.2 × 10^6^ per sample	Pathogen detection by reference-based analyses targeting STEC, molecular serotypes, virulence genes and SNPs complemented with phylogenetic analyses. Microbial community analysis using a *k*-mer approach for identification of bacterial species, phylogroup or serovar	Leonard et al., 2016
**VIRUSES**						
Viral community	Lettuce: 42 field grown samples and 54 retail samples	Pathogen detection and quantification, and characterization of viral community	RNAseq and shotgun metagenome sequencing, complemented with 16S rDNA metataxonomics to verify absence of bacterial DNA on HiSeq2500 (Illumina; 100PE)	Total number of reads approximately 1.5 × 10^9^ (approximately 2.3 to 3.4 × 10^7^ reads per sample)	Assembly of reads followed by mapping to viral reference genomes for taxonomic classification, including detection of putative human pathogens	Aw et al., 2016
Viral community	Fresh parsley plants irrigated with fecally tainted water + water samples and negative controls	Pathogen detection and quantification, and characterization of viral community	RNAseq on MiSeq (300PE)	From 1.2 × 10^6^ (irrigated parsley), to 8.1 × 10^6^ (negative control plants) reads per sample	Assembly of reads followed by mapping to viral reference genomes for taxonomic classification, complemented with phylogenetic analysis	Fernandez-Cassi et al., 2017
Norovirus	Commercial shellfish	Pathogen detection and typing to assess diversity	RNAseq on HiSeq (100PE) and metataxonomics (capsid VP1 gene) on MiSeq (300PE)	Approximately 2 × 10^7^ reads per sample (RNAseq) and from 2 × 10^5^ to 4 × 10^5^ reads per sample (metataxonomics)	Mapping of reads to viral reference genomes for taxonomic classification and genotyping	Imamura et al., 2016b
Norovirus	Farmed oysters	Pathogen detection and typing to assess the performance of a commercially applied decontamination system	Metataxonomics (capsid VP1 gene) on MiSeq (300PE)	From 2 × 10^5^ to 4 × 10^5^ reads per sample	Assembly and mapping to norovirus reference genomes for genotyping	Imamura et al., 2016a

a *The examples are further described and discussed in the main text of this review*.

b *151PE refers to the use of paired-end sequencing with a read length of 151 bp*.

c *STEC, shiga-toxin producing E. coli*.

Jarvis et al. (2015) aimed to characterize the microbiota in cilantro (coriander leaves), and simultaneously identify *Salmonella* from the samples (Table 3). Metataxonomics based on sequencing of the 16S rDNA from 91 samples was complemented with shotgun metagenomics. Gram-negative Proteobacteria dominated in the cilantro samples before enrichment. After 24 h of enrichment the microbial composition had shifted to mainly Gram-positive Firmicutes, as described above for tomato (Ottesen et al., 2013). These findings suggest that the culture-based method should be optimized for the detection of the organisms of interest. Low detection of *Salmonella* by metataxonomics was thought to be due to low sequencing depth and/or reduced amplification efficiency caused by imperfect match in one of the primers. Shotgun metagenomics was performed on six cilantro samples culture-positive for *Salmonella* (enriched samples), and variable levels of *Salmonella* were identified. The genomes of the *Salmonella* isolates from these samples were already fully sequenced and were therefore included in the reference database used in the similarity analysis. The variable levels of *Salmonella* detected after 24 h enrichment illustrate the challenge of detecting *Salmonella* in matrices with a complex microbial background. Again, the sequencing depth of the analysis and levels of contamination were reported to influence the ability to detect the suspected agent.

Similarly, predominance of other species than *L. monocytogenes* was observed until the end of the enrichment procedure (after 40 h) in a study on *L. monocytogenes* and associated microbiota in naturally contaminated ice cream (Ottesen et al., 2016; Table 3).

Leonard et al. (2015) applied shotgun metagenomics to detection of STEC in bagged spinach (Table 3). Spiked samples with known concentrations of a STEC O157:H7 were sequenced and sufficient coverage of the genome required spiking with at least 10,000 colony forming units (CFU) of STEC per 100 g spinach followed by enrichment for 5 h to enable full pathogen characterization. However, enrichment for 23 h allowed the full pathogen characterization by shotgun metagenomics from as little as 10 CFU of STEC spiked into 100 g of spinach. Then, the sequencing coverage of the STEC strain was 184 × and the consensus sequence after reference-based assembly covered the whole reference genome with only six gaps. However, reliable detection to low levels like 10 CFU of STEC per 100 g of spinach, required enrichment for at least 8 h. Then, approximately 2.9% of the reads could be mapped to the reference genome with coverage of approximately 10×. Leonard et al. (2015) concluded that this should be sufficient to enable DNA sequence-based determination of the serotype and essential virulence genes of the contaminating pathogen.

The same team demonstrated the possibility to detect and identify STEC down to strain-level in spinach samples spiked at 10 CFU/100 g of spinach using a variety of STEC strains (Leonard et al., 2016; Table 3). A shotgun metagenomics approach as described in the abovementioned study (Leonard et al., 2015) was applied. For microbial community analysis a database of unique 25-mers for species identification was used. This *k*-mer approach could also differentiate between *E. coli* phylogroups and demonstrated presence of more than one *E. coli* phylogroup in some of the samples. Conserved chromosomal *E. coli* genes (2,542) were extracted from WGS data from the STEC strains used in the spiking experiment as well as the metagenomic assemblies for whole genome phylogeny and SNP analysis. When the metagenomic assemblies only included the spiked STEC strain or the abundance of other *E. coli* was much lower than the spiked strain, the number of mismatches from the SNP analysis was less than 20, i.e., at or close to the intra-strain SNP-variability level (Figure 5).

The above-mentioned studies indicate that at present, it is difficult to achieve the large number of reads required for ≥1 × coverage. These studies also showed how enrichment will bias the microbial composition, potentially favoring other taxa or strains than those intended for enrichment. Since multiple samples often need to be analyzed, it would be cost-efficient if < 1 × coverage would be sufficient for most samples. *K*-mer approaches to screen samples after a minimum of enrichment (see e.g., Ondov et al., 2016) to classify samples according to risk of presence of FBP, could be used to reduce the number of samples for which more in-depth sequencing and analysis is needed. Significant correspondence between a mapped read and FBP specific reference sequences may also provide enough evidence even at < 1 × coverage (Spilsberg et al., 2017). Better sample preparation and enrichment protocols could also contribute. Results from metataxonomics and shotgun metagenomics could aid in optimization of such protocols.

### Viral food pathogens

Complete genome assemblies for 7,409 viruses were available from NCBI on August 16th 2017 (an increase of nearly 500 in 7 months). Viruses lack the genes necessary to transcribe protein-coding genes and replicate and reproduce, and therefore depend completely on the biological machinery of their host cells (Moreira and Lopez-Garcia, 2009). They are highly diverse and lack a common genetic constitution such as genes coding for ribosomal RNAs (Moreira and Lopez-Garcia, 2009), limiting the metataxonomic options. Some viruses can be cultured, but cultivation require advanced protocols, suitable host cells for propagation, and is time-consuming (Rodríguez-Lazaro et al., 2012). Viruses play a dual role in food pathogenesis. Some viruses like bacteriophages can affect the virulence and population structure of microorganisms (Hayes et al., 2017). Other viruses can be FBPs themselves (Newell et al., 2010; EFSA, 2011). For a virus to be transmissible through foods, it must have some environmental stability and remain infectious for some time on or in a food matrix. As viruses are unable to replicate in the food matrix they must have a low infectious dose. Foodborne viruses are commonly shed in large amounts by a fecal route and viral contamination of foods is primarily via human fecal material (Rodríguez-Lazaro et al., 2012). Normal cooking or frying inactivates viruses. Food sources of infections are typically raw foods like fresh produce, soft berry fruits, herbs, shellfish, ready-to-eat products in general and undercooked meat or foods served cold that are contaminated by an infected food-handler post cooking (Halliday et al., 1991; Hedberg and Osterholm, 1993; de Wit et al., 2003; Fiore, 2004; EFSA, 2011, 2015a). Most viruses that can infect through a foodborne route can also utilize a person-to-person infectious route. Many outbreaks, potentially the majority, are simultaneously propagated via combined person-to-person and foodborne infectious routes. Food and water associated transmission is also suspected to enhance the spread of zoonotic viruses and facilitates the occurrence of zoonotic events, e.g., through the handling of bushmeats (Nieuwenhuijse and Koopmans, 2017 and refs. therein). The viral FBP load is often low and heterogeneously dispersed in the food matrix while it is high in clinical patients. It is not trivial to distinguish between foodborne and person-to-person infections, unless initial cases are identified and analyzed. The reporting and surveillance of foodborne viruses is limited, and disease symptoms can be very similar for some viruses and for other viruses differ substantially between infected individuals. All this results in low documentation of the real impact and diversity of foodborne viruses (Nieuwenhuijse and Koopmans, 2017).

The most notable FBP viruses are norovirus (NoV), hepatitis A virus (HAV) and hepatitis E virus (HEV) which are all positive-sense, single-stranded, non-enveloped RNA viruses, and the double-stranded RNA rotavirus (RV) (Newell et al., 2010; EFSA, 2011). Severe acute and Middle East respiratory syndrome (SARS and MERS) and Ebola viruses are examples of other (zoonotic) RNA viruses suspected to be transmissible via food (Newell et al., 2010; Nieuwenhuijse and Koopmans, 2017 and refs. therein). Most FBP viruses are RNA viruses and require special sample processing and nucleic acid extraction methods in contrast to the vast majority of bacteriophages, which are double-stranded DNA viruses.

Viruses, and in particular RNA viruses, evolve rapidly, both via genetic drift and in response to active selection pressures (Holland et al., 1982). Detection and genotyping with PCR approaches, including metataxonomic HTS approaches, can therefore fail. Particular sample processing steps can significantly improve the probability of detection, e.g., by concentration of the viruses and/or removal of interfering substances [reviewed in Hartmann and Halden (2012); see also (EFSA, 2011)]. Detailed understanding of virus stability, inactivation times and temperatures is lacking due to limitations in model systems (Cook, 2013).

#### Outbreak investigations

Isolates of viruses are rare in clinical settings, but clinical samples from viral outbreaks can contain high titers of the causative virus strain(s). The starting points for outbreak investigations are therefore availability of clinically derived samples. Due to the relative heterogeneity of samples compared to isolates, the analytical approaches derive from metagenomics approaches. The small genome size and high mean coverage per nucleotide, however, usually allows for characterization at strain level.

The severity of HAV infection varies strongly with age. HAV is endemic in regions with inadequate sanitation and limited access to clean water, creating population wide immunity. Contrastingly, regions with high quality sanitation and water supply can experience outbreaks of hepatitis A unless broad scale vaccination has been performed. Nearly 300,000 persons were infected in a clam-related epidemic of HAV in Shanghai in 1988 (Halliday et al., 1991) while 1,589 were reported infected, including 2 casualties, in a recent outbreak in Europe associated with frozen berries (Severi et al., 2015). In a recent epidemiological case study, a single food product was associated with a HAV outbreak (Collier et al., 2014; Table 2). HAV was extracted from serum and fecal samples from 120 patients. Metataxonomics targeting a 315 bp HAV fragment yielded 117 (98%) positive for HAV genotype IB. Of these, 99 (85%) were identical in the 315 bp sequenced segment. Attempts to isolate HAV from the food product (frozen pomegranate arils imported from Turkey) were unsuccessful. This demonstrates the challenge of establishing etiologies. Chiapponi et al. (2014) analyzed two samples of frozen berries from two apparently unrelated HAV outbreaks in Italy, and successfully detected HAV by reverse transcription, quantitative PCR (Table 2). RNA was then extracted from the two samples and complete sub-genotype IA HAV genomes of 7,398 and 7,393 nucleotides, respectively, were obtained by a combined RNAseq and amplicon HTS strategy. Chiapponi et al. were able to link the two outbreaks and also link the food derived sequences to an existing patient derived sequence by ≥99.9% nucleotide identities.

RV primarily infects children, is the most common source of gastroenteritis among infants (Desselberger, 2014) and is estimated to cause approximately 5% of total child deaths worldwide. An outbreak of foodborne gastroenteritis in Japan in 2012 caused by RV was probably associated with consumption of raw sliced cabbage (Mizukoshi et al., 2014; Table 2). Samples from patients and food handlers were positive for RV. The study combined a broad spectrum of tests. One clinical, fecal sample was subjected to RNAseq. Sequences of all 11 segments of the viral genome, sufficient to determine the specific viral strain, were identified. No food, however, was assessed for presence of pathogens.

HTS has accelerated the study of viral genetics, the assembly of novel viral genomes, and molecular epidemiology of viral outbreaks (Finkbeiner et al., 2009; Kundu et al., 2013; Wong et al., 2013; Smits et al., 2014; Ganova-Raeva et al., 2015). Studies of virus variants and quasispecies, analyses of vaccine escapes and drug resistance and viral evolution are now almost exclusively performed with HTS (Barzon et al., 2011).

The etiology of virus-associated outbreaks will often remain unknown, for various reasons. Finding and characterizing the causative virus may not be included among the analytical objectives, or the applied method is not always sufficiently sensitive. Other reasons for failure can be genetic drift in the virus, or an outbreak caused by a novel virus. Discovery of pathogens almost exclusively starts with samples from a diseased hosts and not a food matrix. There are many examples of investigations unraveling the etiology of viral outbreaks, but very few where the source of infection is identified or a specific food suspected and tested. It is difficult to establish if an outbreak started as a food contamination event, and it is reasonable to assume that these events are underreported. The small genome sizes of viruses may partly explain why the massive capacity of HTS is rarely used in viral FBP outbreak investigations, as may the limited availability of effective enrichment methods. The potency of new HTS platforms to provide valuable epidemiological information to large viral outbreaks was recently demonstrated by Quick et al. (2016), although on clinical isolates. A similar approach could be applied to epidemiological monitoring and source identification in case of a foodborne outbreak.

#### Surveillance and control purposes

The viral titers in food products are commonly much lower than in clinical samples, and isolates are not available. As for outbreak investigations the approaches applied for surveillance and control are metagenomics derived while the end-point is strain characterization.

Aw et al. (2016) used assembly of RNAseq and shotgun metagenomics reads (mean contig size 680 bp) and mapping to a database of viral reference genomes on samples from field grown and retail lettuce (Table 3). A small fraction of the reads corresponded to RV and other viruses that infect humans. In the study, 16S rDNA metataxonomic screening was used to verify absence of contaminating bacterial DNA.

Fernandez-Cassi et al. (2017) used RNAseq and mapping to examine the viral contamination of fresh parsley plants irrigated with fecally tainted river water (Table 3). A small fraction (< 1%) of the reads was related to FBPs, including among others HEV and NoV.

NoV is extremely contagious, and can cause large outbreaks of gastroenteritis (de Wit et al., 2003). NoV is the no. 1 cause of diarrheal disease and mortalities in the world (Pires et al., 2015) and the leading source of foodborne illness in the USA (Scallan et al., 2011). The etiology is reviewed elsewhere (Moore et al., 2015). Imamura et al. (2016b) used HTS to characterize NoV diversity in shellfish from two commercial producers in Japan, using a combination of RNAseq on virus suspensions and PCR enriched targeted sequencing (genotyping approach similar to metataxonomics; Table 3). NoV genotypes GI.3 and GI.4 were most prevalent and identified in a surprisingly high proportion of 20–25% of the samples. This proof of concept study could not actually address the diversity of NoV in single shellfish as 3 individuals were pooled to one sample prior to analysis to obtain sufficient starting material. The same genotyping approach was applied to a study of the efficiency of removing NoV from shellfish by depuration (Imamura et al., 2016a; Table 3). Depuration is used to decontaminate commercial shellfish. The study demonstrated that depuration is insufficient with respect to NoV.

### Fungal food pathogens

On August 16th 2017, there were 2,515 genome assemblies and 29 complete fungal genomes available from NCBI. So far, there are very few examples of application of HTS to studies of epidemiology and virulence of fungal FBPs (Billmyre et al., 2014; Lee et al., 2014; Litvintseva et al., 2014, 2015; Vaux et al., 2014). Only two published studies relate to specific cases of fungal food pathogenesis (Lee et al., 2014; Vaux et al., 2014). Molecular typing of fungal isolates and mycobiotas in relation to FBD is almost exclusively metataxonomic, but usually limited to PCR amplification of one or a few genetic loci followed by Sanger sequencing with exceptional examples of MLST analysis in the published literature (e.g., Byrnes et al., 2010; Desnos-Ollivier et al., 2015; Wang et al., 2015c). The relevance of fungi as FBPs is debated, significantly lower than that of bacteria and viruses, but possibly also understudied.

Plants are commonly infected in the field by “field fungi.” Some of these produce toxic metabolites and may consequently cause disease when plant derived products are consumed (Lee et al., 2015; Stoev, 2015). Typical examples are *Fusarium* spp. on cereal grains, producing zearalenone, fumonisins and trichothecenes (e.g., deoxynivalenol, T-2 and HT-2 toxin), and *Penicillium* spp. on fruits, producing patulin. Other fungi infect a broad range of food and feed products during storage (“post-harvest fungi”). Typical examples are *Aspergillus* spp. and *Penicillium* spp., producing acute or chronically toxic compounds such as aflatoxins, ochratoxins and citrinin, and multiple antimicrobials with indirect health effects via modulation of the gut microbiota (Gillings et al., 2015; Stoev, 2015). Some mycotoxins are persistent to food processing (EMAN, 2015). They can exacerbate the infections with a wide range of non-fungal and fungal pathogens (Antonissen et al., 2014; Stoev, 2015), and it is hypothesized that mycotoxins may contribute to fungal infections (mycoses; Withlow and Hagler, 2016).

Several fungi are opportunistic, infective FBPs (Clemons et al., 2010; Iriart et al., 2010; Gurgui et al., 2011; Kazan et al., 2011; Benedict et al., 2016). Invasive fungal infections are primarily a problem in immunocompromised people (Brown et al., 2012; Bitar et al., 2014; Benedict et al., 2016). Reported examples of verified foodborne fungal infections are sparse (Benedict et al., 2016), and the problem is perhaps under-investigated. Hitherto, there is only one example of the use of HTS to investigate a fungal FBP outbreak. We suspect that the investigation of several other outbreaks or sporadic cases of fungus associated FBD reported in the literature (Benedict et al., 2016) could have benefited from application of HTS approaches similar to those described for other FBP taxa in this review.

#### Outbreak investigation

As with bacteria the starting points for fungal foodborne outbreak investigations are usually clinically derived isolates. Both strain characterization and metagenomics approaches can be used and are described in the literature, but only one published example applied HTS.

Mucoralean fungi cause mucormycosis (zygomycosis), fatal fungal infections in humans whose incidence has been increasing lately (Roden et al., 2005; Spellberg, 2012). Gastrointestinal mucormycosis is rare and thought to be secondary to ingestion of fungi (Roden et al., 2005). In 2013 a strain of *Mucor circinelloides* f. *circinelloides*, the most virulent subspecies of *M. circinelloides*, was found as a contaminant in a batch of yogurt in the USA (FDA, 2013). More than 200 consumers became ill, although no fatalities were recorded (Lee et al., 2014). The affected consumers were immunocompetent. An isolate obtained from a yogurt container was subjected to WGS in order to characterize its genetic potential to cause significant infections, and to establish its genetic relationship to other strains of *M. circinelloides* (Lee et al., 2014; Table 2). A reference genome assembly was obtained from a strain of *M. circinelloides* isolated from human skin (Findley et al., 2013). Reads from the yogurt isolate were mapped to the reference genome for SNPs analysis. Comparison with a third isolate of a *Mucor* sp. using whole-genome alignments, and pathogenicity studies in murine models contributed to verify the pathogenicity of the yogurt strain. No clinical isolate from the outbreak was included in this study.

### Parasitic food pathogens

Parasites are a diverse (polyphyletic) group of small animals or animal-like Eukaryotes, and their pathogenic potential is less frequently linked with metabolites and more frequently with their energy consumption and predation on host tissues than bacteria and fungi. World-wide, more than 100 species of foodborne parasites cause disease in humans (Orlandi et al., 2002). Complete or draft genome assemblies were available from NCBI for at least 40 of these species on August 16th 2017. Globalization, i.e., the movement of people, animals and food and feed increases the risk of moving and spreading parasites that are originally endemic, to new countries and hosts (Robertson et al., 2014). The lack of suitable enrichment methods for parasites, as opposed to most of the known bacterial and fungal FBPs, means that recovery/isolation steps are particularly important (Robertson et al., 2014). This also suggests that molecular detection can be useful to monitor presence, distribution and epidemiology, as well as the efficiency of clinical treatments after parasite infections.

Published studies applying HTS technologies to parasites are with few exceptions limited to characterization of genomes and transcriptomes, a topic not covered here. These studies, however, provide for detailed insight into the genetics of adaptations to specialized parasitism, and provide urgently needed reference sequence data for detection and identification purposes and clues to possible target/drug combinations (e.g., Tsai et al., 2013; Foth et al., 2014; Young et al., 2014; Barratt et al., 2015).

The presence of a multitude of other taxa and the frequently low abundance of parasites or derived DNA in clinical fecal samples challenge the detectability and may prevent effective sampling and purification of DNA for detection of parasites. Specialized protocols may be required to purify and enrich the parasite relative to a background matrix such as food or feces. Improved sample preparation and enrichment methods are discussed later.

Parasites are Eukaryotes with substantially more genetic similarity to their human and animal hosts than Bacteria. The size of parasite genomes (10–1,000 Mbp) ranges from 10 × the size of bacterial genomes and up to nearly the size of the human genome. New databases are in development, collecting genomic information for various parasites (Martin et al., 2015). The lack of annotated genomes and transcriptomes has been a major obstacle to the effective use of HTS for detection of parasites. Molecular discrimination is dependent on detailed knowledge of genomes and genetic variation. However, many of the parasites are detectable by visual, macro- or microscopic inspection, and this is likely to be a more cost-efficient approach in many instances.

#### Outbreak investigation

The intraspecific variation in virulence among foodborne parasites is, to our knowledge, not reported to be high or significant. Combined with the large genome size of Eukaryotes, this suggests that metataxonomic approaches can be sufficient for detection and outbreak investigations.

More than 200 seafood poisoning cases of unknown etiology were reported in Japan from 2008 to 2010. Victims commonly reported to have ingested raw *Paralichthys olivaceus* (a flounder). Kawai et al. (2012) therefore extracted total DNA or RNA from frozen *P. olivaceus* filets for shotgun metagenomics and RNAseq (Table 2). In parallel, muscle tissue was sieved to recover spores from suspected parasite infections. The presence of spores and 18S rDNA from *Kudoa septempunctata* in the fish samples was observed. The pathogenicity of this myxosporean was confirmed in suckling mice and house musk shrews.

### Vectors of foodborne pathogens

In addition to pathogens naturally associated with the food producing organisms such as gut microbes, dermal yeasts and bacteria, plant pathogenic fungi, etc. many of the most severe food pathogeneses are caused by incidental transfer from animal vectors (pests; Olsen et al., 2001; Jones et al., 2013). The Food and Drug Administration has identified the 22 most common pests contributing to the spread of FBD in the USA (Olsen et al., 2001). Four of these are rodents (mouse and rat species), while the remaining 18 are insects (cockroaches, ants and flies). The traditional approach to detect and identify these is microscopy. Recently it was proposed to use metataxonomics by sequencing of the mitochondrial cytochrome c oxidase subunit I (COI) as a faster, more reliable, sensitive and cost-efficient approach (Jones et al., 2013). COI metataxonomics can be performed using HTS (see Figure 4) and may therefore suit as an attractive approach to screen routinely for presence of these vectors in raw materials for food production, to reduce the risk of introducing pathogens in the production. Vectors other than, or in addition to the 22 identified by Olsen et al. (2001) may be identified as particularly relevant, e.g., in other parts of the world. The introduction of additional sequence targets in an HTS based metataxonomic screening should be feasible (Lammers et al., 2014; Leray and Knowlton, 2015; Arulandhu et al., 2017) despite the limitations of COI and metataxonomic approaches in general (Deagle et al., 2014; Staats et al., 2016; Arulandhu et al., 2017).

### Other applications of HTS with potential relevance to FBPs

The discovery and characterization of biosynthetic gene clusters in microorganisms is radically facilitated with the availability of HTS (Cacho et al., 2015). Secondary metabolites are often toxic, and many of the microorganisms producing them are FBPs. Characterization of the biosynthetic pathways provides a basis for faster detection of agents producing the metabolites, as well as for interception of undesirable biological effects and exploitation of the biosynthetic pathways for production of new bioactive compounds.

Direct shotgun metagenomics on DNA purified from microorganisms isolated without enrichment culturing is an attractive approach. Such an approach applied to clinical, polymicrobial urine samples was found to have comparable identifiability of bacteria as more conventional and much more time consuming approaches (Hasman et al., 2014). The complexity of such urine samples may be comparable to that of some food matrixes. Several approaches to data analysis were taken. First: identification of microorganisms by presence of specific *k*-mer motifs identified in a database of complete bacterial genomes. Secondly: alignment based subtraction of host-DNA derived reads and estimation of relative bacterial species distribution. Third: mapping of reads against a larger database of complete and draft bacterial, archaeal, fungal, protozoan and viral genomes. Finally, MLST and resistance gene identification performed against relevant databases. In a follow up study, direct shotgun metagenomics on toilet waste samples from long-distance flights was used to identify bacteria and antimicrobial resistance genes (Petersen et al., 2015) by mapping of reads to the abovementioned databases.

Host-specific genetic markers may be present in strains of pathogenic and non-pathogenic taxa, and then hold potential for source tracking (Gomi et al., 2014). See also Franz et al. (2016) for a more detailed discussion on source attribution.

## Discussion

### Acknowledging the importance of sample preparation

A major challenge for detection of FBPs in complex matrixes is the recovery of the organism or its genes. Foods are physically and chemically complex matrices hosting complex microbial communities. The extraction and purification of DNA from the samples is of major importance as the presence of inhibitors may hamper the analysis further down the line (Ceuppens et al., 2014; Moore et al., 2015). The sample storage conditions and preparation steps can introduce taxonomic biases linked to recovery (Ceuppens et al., 2015; Menke et al., 2017). Similar or even stronger effects on the microbiome and RNA population of samples are predictable, as also the intraspecific diversity and gene expression can be affected. The size of isolated/purified nucleic acid fragments is also relevant. Longer fragments are usually superior and complementary to shorter fragments for genome assembly. The purity and relative concentration of target isolated nucleic acids affect their detectability and certainly their quantifiability (Holst-Jensen et al., 2003). Different taxa and life stages of potential pathogens require different treatments for detachment from substrates and complex structures, recovery and lysis. Protocols required for extraction and purification of DNA from strongly attaching, biofilm-forming taxa with tough cell walls can cause shearing of nucleic acids of other relevant taxa, yielding undesirably short fragments or totally degraded RNA/DNA. The stability of nucleic acids is a critical parameter, and this topic is reviewed by Ceuppens et al. (2014). Enrichment processes required to achieve the necessary target concentration can also bias the post-enrichment microbial composition in undesirable ways, as illustrated with *Salmonella* spp. in examples discussed earlier (Ottesen et al., 2013; Jarvis et al., 2015).

Examples of non-culturing based enrichment of particular organisms include size based filtering, buoyancy, affinity based columns or immuno-magnetic separation (Hadfield et al., 2015). Molecular enrichment can be achieved by subtraction hybridization (Galbraith et al., 2004) and various combinations of digestion with restriction enzymes, adapter ligation and sequence specific amplification (Leichty and Brisson, 2014; Arulandhu et al., 2016).

### Specific challenges related to molecular analyses

Ability to discriminate viable/infective agents from dead/non-infective agents and to obtain isolates of the agent(s) from food samples for comparison purposes is often important (Ceuppens et al., 2014; Forbes et al., 2017). Molecular analytical methods can potentially circumvent need for enrichment culturing and further selective steps for the detection of many of the most important FBPs, but the issues of viability, recovery and LOD remain critical. Transcriptomics or use of propidium monoazide prior to sequencing are options to discriminate living from dead cells (Weinmaier et al., 2015). There is no harmonized requirement for detection and/or identification of an FBP by HTS, neither with respect to the number of reads, coverage of a specific diagnostic target motif, minimum length of (assembled) contig or maximum number of mismatches. Depending on the choice of actual minimum performance parameters and associated acceptance values it is possible to calculate the probability of detection (POD) of relevant target(s) and perform a statistical comparison of the POD to the actual observations (Holst-Jensen et al., 2016; Spilsberg et al., 2017). This would provide clues to the probability and reliability of findings. POD calculations performed prior to analyses can be useful to assess the cost-efficiency of alternative approaches. HTS is generally not quantitative, and complementary tools are needed to verify the LOD and recovery. Direct sequencing of nucleic acids from a clinical or food derived sample can be faster than culture-dependent analytical approaches. However, successive analysis of HTS data, necessary for interpretation, may be too time-consuming to render HTS really competitive in many cases. For bacteria in particular, the combination of limited enrichment culturing and fast HTS with (semi-)automated bioinformatics is currently the most optimal, realistic option that can provide very detailed information while simultaneously offering sufficient sensitivity and speed. For those FBPs that cannot be enriched by culturing, future pipeline developments may improve the situation, at least for certain types of matrixes and scenarios.

### Harmonization and validation of HTS approaches

The use of different technologies and methods negatively affect the comparability of results (Junemann et al., 2013), as does lack of harmonized terminology and data interpretation (Lambert et al., 2017; Taboada et al., 2017). Recent studies have tried to overcome this variability in end-point results by establishing standard WGS data sets from outbreaks with *Salmonella*, STEC and *L. monocytogenes* (Timme et al., 2015) and a benchmarking dataset consisting of 101 whole genome sequences from one *E. coli* hypermutator strain (Ahrenfeldt et al., 2017). Use of these data sets is proposed to facilitate standardization and harmonization of bioinformatics pipelines (Timme et al., 2015; Ahrenfeldt et al., 2017).

Traditional approaches to standardization and validation cannot be directly applied to HTS approaches. There is for example no common cognition as to what is required for “identification” of a FBP, such as the number of organism-specific reads (positive calls), sequence depth (confidence), LOD, etc. for each platform and protocol, or number of differences distinguishing isogenic from non-isogenic isolates (Figure 5). There are, however, several efforts to improve the situation. Guidelines for harmonization of clinical testing by application of HTS have been published and may provide useful guidance also to other sectors including FBP detection (Gargis et al., 2012, 2015; Weiss et al., 2013; Aziz et al., 2015). The Global Microbial Identifier (GMI) was initiated in 2011 (GMI, 2011; Aarestrup et al., 2012). The background for this initiative was the high number of microbiological isolates that are characterized annually with very diverse and expensive typing systems, the increasing number of infectious diseases with global epidemiology requiring rapid detection and identification of microbial agents, the likelihood that microbiological laboratories (primarily clinical) will have DNA sequencers available in the foreseeable future, and that the likely future limiting factor is not the HTS cost but the assembly, processing and data handling in a standardized way to make the information useful (GMI, 2011). In 2015 the GMI launched the first HTS proficiency testing scheme in this respect (GMI, 2015; Moran-Gilad et al., 2015). Recent progress by GMI is reported by Taboada et al. (2017). The World Organization for Animal Health also recently launched its first standards for HTS, bioinformatics and computational genomics (OIE, 2017). Two other examples are the International Organization for Standardization's initiative to standardize WGS for typing and genomic characterization (ISO/TC 34/SC 9/WG 25) and the initiative to standardize the format of HTS derived SNP data (in a cancer context; Pipan and Kunaj, 2015), respectively.

### Results interpretation

HTS can generate a vast amount of data describing the microbial community of foods. When technical issues such as speed and standardization of laboratory and bioinformatics methods are improved, HTS and especially shotgun metagenomics may find applications in routine analysis of food, also for control purposes, as it has already done in clinical settings. Interpretation of the results is an important issue, for both the agri-food industry and the competent authorities, but also how to act on the results (Lambert et al., 2017; Taboada et al., 2017).

In the quality control of products and production lines, the detection of a pathogen, a genus that includes pathogenic species, and/or specific virulence genes/factors may, but will not necessarily lead to withdrawal of the product or lead to decisions to decontaminate the entire production line. The decision will depend on the specific context, i.e., the specific combination of pathogen marker, how well characterized the pathogen is, the type of product and the intended use. Quantitative data may also play a role, but sometimes are not or cannot be made available. Legal requirements can direct the decision processes. In the European Union (EU), both food safety criteria and process hygiene criteria are listed with details on sampling plans, limits, analytical reference methods and stage where the criterion applies (European Commission, 2005). Similarly, the safety of the food supply chain in the USA is regulated (FDA, 2011).

The application of WGS in microbiological risk assessments of foods is largely unexplored and faces important challenges. The number of hazards increase exponentially when zooming from serovars or serotypes to genotypes. The translation of multidimensional genotypic data into reduced information on phenotypes to ultimately generate a measure of risk that matches the requirements of food safety authorities and policy makers is therefore necessary (Franz et al., 2016). The presence or absence of specific genetic markers in a bacterial isolate or in a sample can direct the acceptance or rejection of a particular product. Furthermore, specific detection of FBP-markers may only be required within a narrow concentration range. For example, the observed presence of the STEC-associated virulence genes *stx1* or *stx2* in ground beef can lead to rejection or at least to particular caution and further examination to preclude the presence of STEC. We suggest that the observed background level of *E. coli* can provide a basis for decision in the case of STEC. The presence of *E. coli* itself is undesirable and un-tolerable background levels of *E. coli*, e.g., ≥ 10^4^ CFU g^−1^ should lead to rejection. Thus, we believe it is most critical to ensure that the POD for STEC (markers) is acceptable within the entire range of concentrations at tolerable background levels, e.g., POD ≥ 99% at < 10^4^ CFU *E. coli* g^−1^. We suggest that such an approximation can be used to guide both method developments and the enrichment efforts needed to obtain conclusive data. The infectious dose of FBPs vary, and for some FBPs it can be < 100 cells (Tilden et al., 1996; Tuttle et al., 1999; Hall, 2012) and must be considered for this type of calculation.

Recently, it has been questioned if all protists and helminths, historically thought of as “parasites” are indeed parasites (Lukes et al., 2015). Growing evidence of the diverse functional roles that specific bacteria can take on in humans, has forced us to acknowledge that beneficial and pathogenic may be two sides of the same organism, and that it must be understood in the broader contexts of ecology, symbiosis and diversity, among others. Collecting data on the entire microbial communities associated with their hosts may provide for better understanding of the functional interplay between hosts and hosted microorganisms, though so far no clear trend is emerging (Martin et al., 2015). In the future, perhaps, by learning more aided by HTS studies, it will be possible to stimulate beneficial and prevent pathogenic behavior by microorganisms with broad phenotypic potentials.

### Capacity challenges

#### Low sequencing costs, but high costs overall

HTS has profound impacts on both academic research and practical diagnostic and clinical surveillance. Despite this, there are several obstacles to its full adaptation in the routine detection of FBPs. The ideal HTS solution for the detection of FBPs should be rapid, accurate, operable, and economic. Obstacles include the purchase and implementation of the HTS platforms, but perhaps more importantly the subsequent data analysis and results interpretation (Loman et al., 2013; ECDC, 2015b). Sequencing costs have declined exponentially in the past decade, a development expected to continue. However, also costs associated with sample collection and preparation, nucleic acid extraction and purification, as well as any other steps in the preparation of sequencing libraries, sequencing, data management and downstream analyses must be considered (Sboner et al., 2011). As long as the rapid decline in the cost of data generation is not matched by a corresponding reduction in data storage, maintenance and processing costs, there will remain a substantial economic barrier preventing the full implementation of HTS in research and routine for FBP identification.

#### Computational capacity is a major hurdle

HTS requires large computational capacities, i.e., various tools for data storage and data analysis, including data management, quality control, mapping and alignment, *de novo* assembly, scaffolding, gene annotation, metagenomics and biological significance interpretation (Cuccuru et al., 2014; Mayo et al., 2014). These bioinformatics tools, particularly their proper usage and output interpretation appear as an impenetrable barrier facing almost all scientists in either fields of food science and microbiology lacking or having weak bioinformatics background. A substantial effort will therefore be required to train relevant staff in use of these tools (Taboada et al., 2017). The application of HTS in FBP research and surveillance is also impeded or slowed down by the complexity of different formats of diverse software tools and limitations of computational capacities (Li et al., 2012; Xia et al., 2012; Cuccuru et al., 2014; Mayo et al., 2014). Several integrative frameworks consisting of publicly available research software and specifically designed pipelines are available for visualizing, querying and downloading the data released, and for HTS data processing and analysis for diverse microorganisms (Li et al., 2012; Wang et al., 2012; Cuccuru et al., 2014; Taboada et al., 2017). We believe it is essential that a corresponding user friendly web-based framework for FBPs detection using HTS is created. To fulfill this purpose, a close and effective collaboration between different scientists from related fields is urgently needed (Stapleton, 2014). These should jointly develop more automatic and reliable bioinformatics tools for sequencing reads' quality control (Edgar and Flyvbjerg, 2015), data compression and extraction (Wang and Zhang, 2011; Lassmann, 2015), data analysis on cloud computation (Kwon et al., 2015), and downstream analysis (Davis et al., 2015; Gweon et al., 2015; Müenz et al., 2015; Ratan et al., 2015; Wang et al., 2015a).

#### Database limitations on access and contents

The establishment of a reference database for each major FBP species or subspecies poses yet another challenge. An accurate and complete reference database for all major FBPs is fundamental to epidemiological investigations and design of pathogen detection assays. The genome sequence is the prerequisite for understanding the molecular basis of a given phenotype of a FBP. Thus, genome sequencing is invaluable in advancing our understanding of virulence mechanisms and epidemiology of FBPs. Many complete or draft genome assemblies of the most common FBPs have been released (Mellmann et al., 2011; Timme et al., 2012; Schmitz-Esser and Wagner, 2014; Quick et al., 2015; Wu et al., 2015). This development is rapidly progressing and open data sharing including uploading of sequence data to public high-quality databases should be encouraged by all stakeholders. The availability of reference genomes is expected to speed up diagnosis and shorten FBD outbreaks, thus promoting public health. Additional complementary efforts are needed for more endemic FBPs.

#### Cost-efficiency of data generation

The massive capacity of HTS can be utilized to find a causative agent or other target of relevance by unbiased ultra-deep sequencing, but this “brute force” approach will generate massive non-target data and, with current sequence pricing, is only applicable to research applications. Late availability of interpretable data can be a drawback of HTS, depending on the protocols used (Loman et al., 2012; Reuter et al., 2013; Quick et al., 2015). Quick et al. (2015) demonstrated (on *Salmonella*) that the time to answer in a hospital outbreak can be reduced significantly without impact on the results using draft shotgun metagenomics by rapid MiSeq sequencing, compared to standard protocols for MiSeq and HiSeq. The same study also demonstrated that samples could be assigned to species level within 20 min, serotype within 40 min and whether the isolate was part of the outbreak in less than an hour using the MinION sequencing technology and a mapping approach. With alignment-free bioinformatics the speed of analysis is further improved (Ondov et al., 2016). Third generation sequencing platforms can report sequences in real-time and this opens the possibility to selectively sequence only the nucleic acid of interest (Loose et al., 2016). That could permit more cost-efficient use of the sequencing capacity. These examples illustrate some recent improvements to the potential of HTS in management of outbreaks.

#### Global imbalance in capacity vs. needs

The populations in developing countries and rural areas are more commonly struggling with FBPs than the inhabitants of major cities in developed, Western countries. The access to advanced analytical technologies is inversely distributed. HTS technologies are only exceptionally developed for point-of-care and in-field applications, but recent examples of outbreak investigations of Ebola and Zika viruses using the MinION (Quick et al., 2016, 2017) indicate that more robust and portable platforms may be available in the foreseeable future. However, even with access to the sequencers, the access to necessary consumables, computer capacities including databases, and competent manpower will likely remain obstacles to the implementation of HTS based analytical methods for many years. This is a serious challenge that must be solved in order to ensure that initiatives to build up global, collaborative networks and systems to cope with emerging FBPs are successful.

## Conclusion

Despite the obvious benefits associated with potential independence from culturing, and the broad spectrum and level of detailed characterization of targets that can be detected simultaneously, we believe that in a short perspective HTS is unlikely to find application outside the most advanced laboratories. The reductionist tradition prevails in many laboratories, i.e., of testing analytically for single agents, in particular in the food sector. Detailed sequencing based characterization of reference strains and representative clinical, food and environmental samples is still in its relative infancy, with many FBP species still practically missing in the databases, and few species where the range of genetic diversity is close to completely characterized. However, as HTS studies accumulate so does the data pool and the appreciation of the potentials of the technology. Third generation HTS technology, as exemplified with the Oxford Nanopore MinION sequencer promise to permit on-site, long-read, real-time sequencing (Mikheyev and Tin, 2014; Greninger et al., 2015; Quick et al., 2015), but still struggles with laborious sample preparation requirements and high error rates. As these obstacles are mitigated, this type of technology will inevitably lead to a paradigm shift in FBP detection.

## Author contributions

CS drafted most of the introduction and section on bacterial food pathogens and contributed to the discussion; AH co-ordinated the manuscript drafting, wrote the sections on fungal and parasitic food pathogens, and vectors and other HTS applications, and contributed to the introduction, discussion and sections on bacterial and viral pathogens; UD, GJ, and WL contributed to the introduction, the section on bacterial pathogens and the discussion; BS drafted the section on viral pathogens and contributed to the introduction and discussion; and WL and JS initiated the review. All authors (CS, AH, UD, GJ, WL, BS, and JS) critically revised the manuscript.

### Conflict of interest statement

The authors declare that the research was conducted in the absence of any commercial or financial relationships that could be construed as a potential conflict of interest.

## References

[B1] Aarestrup F. M. Brown E. W. Detter C. Gerner-Smidt P. Gilmour M. W. Harmsen D.. (2012). Integrating genome-based informatics to modernize global disease monitoring, information sharing, and response. Emerg. Infect. Dis. 18:120453. 10.3201/eid1811.120453 23092707 PMC3559169

[B2] Adeolu M. Alnajar S. Naushad S. Gupta R. S. (2016). Genome-based phylogeny and taxonomy of the ‘Enterobacteriales’: proposal for Enterobacterales ord. nov divided into the families Enterobacteriaceae, Erwiniaceae fam. nov., Pectobacteriaceae fam. nov., Yersiniaceae fam. nov., Hafniaceae fam. nov., Morganellaceae fam. nov., and Budviciaceae fam. nov. Int. J. Syst. Evol. Microbiol. 66, 5575–5599. 10.1099/ijsem.0.001485 27620848

[B3] Ahrenfeldt J. Skaarup C. Hasman H. Pedersen A. G. Aarestrup F. M. Lund O. (2017). Bacterial whole genome-based phylogeny: construction of a new benchmarking dataset and assessment of some existing methods. BMC Genomics 18:19. 10.1186/s12864-016-3407-6 28056767 PMC5217230

[B4] Antonissen G. Martel A. Pasmans F. Ducatelle R. Verbrugghe E. Vandenbroucke V.. (2014). The impact of *Fusarium* mycotoxins on human and animal host susceptibility to infectious diseases. Toxins 6, 430–452. 10.3390/toxins6020430 24476707 PMC3942744

[B5] Arulandhu A. J. Staats M. Hagelaar R. Voorhuijzen M. M. Prins T. W. Scholtens I.. (2017). Development and validation of a multi-locus DNA metabarcoding method to identify endangered species in complex samples. GigaScience 6:gix080. 10.1093/gigascience/gix080 29020743 PMC5632295

[B6] Arulandhu A. J. van Dijk J. P. Dobnik D. Holst-Jensen A. Shi J. Zel J.. (2016). DNA enrichment approaches to identify unauthorized genetically modified organisms (GMOs). Anal. Bioanal. Chem. 408, 4575–4593. 10.1007/s00216-016-9513-0 27086015

[B7] Ashton P. M. Nair S. Peters T. M. Bale J. A. Powell D. G. Painset A.. (2016). Identification of Salmonella for public health surveillance using whole genome sequencing. Peerj 4:e1752. 10.7717/peerj.1752 27069781 PMC4824889

[B8] Ashton P. M. Peters T. Ameh L. McAleer R. Petrie S. Nair S. (2015). Whole genome sequencing for the retrospective investigation of an outbreak of *Salmonella* Typhimurium DT 8. PLoS Curr. Outbreaks 2015:1 10.1371/currents.outbreaks.2c05a47d292f376afc5a6fcdd8a7a3b6 PMC4336196 25713745

[B9] Astridge K. Barker M. Bell R. Combs B. Boyle C. Fearnley E. (2015). Monitoring the incidence and causes of diseases potentially transmitted by food in Australia: Annual report of the OzFoodNet network, (2011). Commun. Dis. Intell. 39, E236–E264. Available online at: http://www.health.gov.au/internet/main/publishing.nsf/Content/cda-cdi3902-pdf-cnt.htm/$FILE/cdi3902g.pdf 10.33321/cdi.2015.39.22 26234259

[B10] Aw T. G. Wengert S. Rose J. B. (2016). Metagenomic analysis of viruses associated with field-grown and retail lettuce identifies human and animal viruses. Int. J. Food Microbiol. 223, 50–56. 10.1016/j.ijfoodmicro.2016.02.008 26894328

[B11] Aziz N. Zhao Q. Bry L. Driscoll D. K. Funke B. Gibson J. S.. (2015). College of American Pathologists' laboratory standards for next-generation sequencing clinical tests. Arch. Pathol. Lab. Med. 139, 481–493. 10.5858/arpa.2014-0250-CP 25152313

[B12] Barratt J. L. Cao M. Stark D. J. Ellis J. T. (2015). The transcriptome sequence of *Dientamoeba fragilis* offers new biological insights on its metabolism, kinome, degradome and potential mechanisms of pathogenicity. Protist 166, 389–408. 10.1016/j.protis.2015.06.002 26188431

[B13] Barzon L. Lavezzo E. Militello V. Toppo S. Palù G. (2011). Applications of next-generation sequencing technologies to diagnostic virology. Int. J. Mol. Sci. 12, 7861–7884. 10.3390/ijms12117861 22174638 PMC3233444

[B14] Benedict K. Chiller T. M. Mody R. K. (2016). Invasive fungal infections acquired from contaminated food or nutritional supplements: a review of the literature. Foodborne Pathog. Dis. 13, 343–349. 10.1089/fpd.2015.2108 27074753 PMC5669373

[B15] Bergholz T. M. Switt A. I. Wiedmann M. (2014). Omics approaches in food safety: fulfilling the promise? Trends Microbiol. 22, 275–281. 10.1016/j.tim.2014.01.006 24572764 PMC4016976

[B16] Billmyre R. B. Croll D. Li W. Mieczkowski P. Carter D. A. Cuomo C. A.. (2014). Highly recombinant VGII *Cryptococcus gattii* population develops clonal outbreak clusters through both sexual macroevolution and asexual microevolution. mBio 5:e01494–14. 10.1128/mBio.01494-14 25073643 PMC4128362

[B17] Bitar D. Lortholary O. Le Strat Y. Nicolau J. Coignard B. Tattevin P.. (2014). Population-based analysis of invasive fungal infections, France, 2001-2010. Emerging Infect. Dis. 20, 1149–1155. 10.3201/eid2007.140087 24960557 PMC4073874

[B18] Blagden T. Schneider W. Melcher U. Daniels J. Fletcher J. (2016). Adaptation and validation of e-probe diagnostic nucleic acid analysis for detection of *Escherichia coli* O157:H7 in metagenomic data from complex food matrices. J. Food Prot. 79, 574–581. 10.4315/0362-028X.JFP-15-440 27052861

[B19] Brown G. D. Denning D. W. Levitz S. M. (2012). Tackling human fungal infections. Science 336, 647–647. 10.1126/science.1222236 22582229

[B20] Brüssow H. Canchaya C. Hardt W. D. (2004). Phages and the evolution of bacterial pathogens: from genomic rearrangements to lysogenic conversion. Microbiol. Mol. Biol. Rev. 68, 560–602. 10.1128/MMBR.68.3.560-602.2004 15353570 PMC515249

[B21] Brzuszkiewicz E. Thürmer A. Schuldes J. Leimbach A. Liesegang H. Meyer F. D.. (2011). Genome sequence analyses of two isolates from the recent *Escherichia coli* outbreak in Germany reveal the emergence of a new pathotype: Entero-Aggregative-Haemorrhagic *Escherichia coli* (EAHEC). Arch. Microbiol. 193, 883–891. 10.1007/s00203-011-0725-6 21713444 PMC3219860

[B22] Byrnes E. J. III Li W. Lewit Y. Ma H. Voelz K. Ren P.. (2010). Emergence and pathogenicity of highly virulent *Cryptococcus gattii* genotypes in the northwest United States. PLoS Pathog. 6:e1000850. 10.1371/journal.ppat.1000850 20421942 PMC2858702

[B23] Cacho R. A. Tang Y. Chooi Y.-H. (2015). Next-generation sequencing approach for connecting secondary metabolites to biosynthetic gene clusters in fungi. Front. Microbiol. 5:774. 10.3389/fmicb.2014.00774 25642215 PMC4294208

[B24] Ceuppens S. Delbeke S. De Coninck D. Boussemaere J. Boon N. Uyttendaele M. (2015). Characterization of the bacterial community naturally present on commercially grown basil leaves: evaluation of sample preparation prior to culture-independent techniques. Int. J. Environ. Res. Public Health 12, 10171–10197. 10.3390/ijerph120810171 26308033 PMC4555336

[B25] Ceuppens S. Li D. Uyttendaele M. Renault P. Ross P. Van Ranst M. (2014). Molecular methods in food safety microbiology: interpretation and implications of nucleic acid detection. Comprehen. Rev. Food Science Food Safety 13, 551–577. 10.1111/1541-4337.12072 33412695

[B26] Chattaway M. A. Dallman T. J. Gentle A. Wright M. J. Long S. E. Ashton P. M.. (2016). Whole genome sequencing for public health surveillance of Shiga toxin-producing *Escherichia coli* other than serogroup O157. Front. Microbiol. 7:258. 10.3389/fmicb.2016.00258 26973632 PMC4776118

[B27] Chiapponi C. Pavoni E. Bertasi B. Baioni L. Scaltriti E. Chiesa E.. (2014). Isolation and genomic sequence of hepatitis A virus from mixed frozen berries in Italy. Food Environ. Virol. 6, 202–206. 10.1007/s12560-014-9149-1 24859055 PMC4119586

[B28] Clemons K. V. Salonen J. H. Issakainen J. Nikoskelainen J. McCullough M. J. Jorge J. J.. (2010). Molecular epidemiology of *Saccharomyces cerevisiae* in an immunocompromised host unit. Diagn. Microbiol. Infect. Dis. 68, 220–227. 10.1016/j.diagmicrobio.2010.06.010 20846806

[B29] Collier M. G. Khudyakov Y. E. Selvage D. Adams-Cameron M. Epson E. Cronquist A.. (2014). Outbreak of hepatitis A in the USA associated with frozen pomegranate arils imported from Turkey: an epidemiological case study. Lancet Infect. Dis. 14, 976–981. 10.1016/S1473-3099(14)70883-7 25195178

[B30] Cook N. (2013). Viruses in Food and Water: Risks, Surveillance and Control. Cambridge, UK: Woodhead Publishing.

[B31] Crim S. M. Iwamoto M. Huang J. Y. Griffin P. M. Gilliss D. Cronquist A. B. (2014). Incidence and trends of infection with pathogens transmitted commonly through food - Foodborne Diseases Active Surveillance Network: 10 US Sites, 2006-2013. MMWR 63, 328–332. Available online at: https://www.cdc.gov/mmwr/pdf/wk/mm6315.pdf 24739341 PMC5779392

[B32] Cuccuru G. Orsini M. Pinna A. Sbardellati A. Soranzo N. Travaglione A.. (2014). Orione, a web-based framework for NGS analysis in microbiology. Bioinformatics 30, 1928–1929. 10.1093/bioinformatics/btu135 24618473 PMC4071203

[B33] Davis S. Pettengill J. Luo Y. Payne J. Shpuntoff A. Rand H. (2015). CFSAN SNP Pipeline: an automated method for constructing SNP matrices from next-generation sequence data. PeerJ Comput. Sci. 1:11 10.7717/peerj-cs.20

[B34] Davis T. K. Van De Kar N. C. A. J. Tarr P. I. (2014). Shiga toxin/Verocytotoxin-producing *Escherichia coli* infections: practical clinical perspectives. Microbiol. Spect. 2:EHEC-0025-2014. 10.1128/microbiolspec.EHEC-0025-2014 26104210

[B35] de Wit M. A. Koopmans M. P. G. van Duynhoven Y. (2003). Risk factors for norovirus, Sapporo-like virus, and group A rotavirus gastroenteritis. Emerging Infect. Dis. 9, 1563–1570. 10.3201/eid0912.020076 14720397 PMC3034344

[B36] Deagle B. E. Jarman S. N. Coissac E. Pompanon F. Taberlet P. (2014). DNA metabarcoding and the cytochrome c oxidase subunit I marker: not a perfect match. Biol. Lett. 10:20140562. 10.1098/rsbl.2014.0562 25209199 PMC4190964

[B37] Desnos-Ollivier M. Patel S. Raoux-Barbot D. Heitman J. Dromer F. Group F. C. S. (2015). Cryptococcosis serotypes impact outcome and provide evidence of *Cryptococcus neoformans* speciation. mBio 6:e00311–15. 10.1128/mBio.00311-15 26060271 PMC4462623

[B38] Desselberger U. (2014). Rotaviruses. Virus Res. 190, 75–96. 10.1016/j.virusres.2014.06.016 25016036

[B39] ECDC (2015a). Annual Epidemiological Reports [Online]. European Centre for Disease Prevention and Control,. Available online at: http://ecdc.europa.eu/EN/PUBLICATIONS/SURVEILLANCE_REPORTS/annual_epidemiological_report/Pages/epi_index.aspx [Accessed 4th November 2015].

[B40] ECDC (2015b). Expert Opinion on the Introduction of Next-Generation Typing Methods for Food- and Waterborne Diseases in the EU and EEA. Stockholm: Sweden: European Centre for Disease Prevention and Control.

[B41] Edgar R. C. Flyvbjerg H. (2015). Error filtering, pair assembly and error correction for next-generation sequencing reads. Bioinformatics 31, 3476–3482. 10.1093/bioinformatics/btv401 26139637

[B42] EFSA (2011). Scientific Opinion on an update on the present knowledge on the occurrence and control of foodborne viruses. EFSA J. 9, 2190–2285. 10.2903/j.efsa.2011.2190 PMC7163696 32313582

[B43] EFSA (2013). Scientific opinion on VTEC-seropathotype and scientific criteria regarding pathogenicity assessment. EFSA J. 11, 3138–3243. 10.2903/j.efsa.2013.3138

[B44] EFSA (2014). Use of Whole Genome Sequencing (WGS) of Food-Borne Pathogens for Public Health Protection. EFSA.

[B45] EFSA (2015a). The European Union summary report on trends and sources of zoonoses, zoonotic agents and food-borne outbreaks in (2013). EFSA J. 13, 165 10.2903/j.efsa.2015.3991 PMC7009540 32625785

[B46] EFSA (2015b). The European Union summary report on trends and sources of zoonoses, zoonotic agents and food-borne outbreaks in (2014). EFSA J. 13, 191 10.2903/j.efsa.2015.4329 PMC7009540 32625785

[B47] EMAN (2015). European Mycotoxin Awareness Network: Basic Factsheet Trichothecenes. European Mycotoxin Awareness Network. Available online at: http://eman.leatherheadfood.com/node/45

[B48] Emond-Rheault J.-G. Jeukens J. Freschi L. Kukavica-Ibrulj I. Boyle B. Dupont M.-J.. (2017). A syst-OMICS approach to ensuring food safety and reducing the economic burden of salmonellosis. Front. Microbiol. 8:996. 10.3389/fmicb.2017.00996 28626454 PMC5454079

[B49] European Commission (2005). Commission Regulation (EC) No 2073/2005 of 15 November 2005 on microbiological criteria for foodstuffs. Official J. Eur. Union 2005, 1–26. Available online at: http://eur-lex.europa.eu/LexUriServ/LexUriServ.do?uri=OJ:L:2005:338:0001:0026:EN:PDF

[B50] FDA (2011). FDA Food Safety Modernization Act, Public Law 111-353-January 4th 2011, 124 Stat. (3885). Washington, DC: United States Government Printing Office; Government of the United States of America.

[B51] FDA (2013). Establishment Inspection Report, Chobani Idaho, FEEI: 3009726115. U.S. Food and Drug Administration Available online at: http://www.fda.gov/ucm/groups/fdagov-public/@fdagov-afda-orgs/documents/document/ucm376634.pdf.

[B52] Fernandez-Cassi X. Timoneda N. Gonzales-Gustavson E. Abril J. F. Bofill-Mas S. Girones R. (2017). A metagenomic assessment of viral contamination on fresh parsley plants irrigated with fecally tainted river water. Int. J. Food Microbiol. 257, 80–90. 10.1016/j.ijfoodmicro.2017.06.001 28646670

[B53] Ferri E. Galimberti A. Casiraghi M. Airoldi C. Ciaramelli C. Palmioli A.. (2015). Towards a universal approach based on omics technologies for the quality control of food. BioMed Res. Int. 2015, 14. 10.1155/2015/365794 26783518 PMC4691458

[B54] Findley K. Oh J. Yang J. Conlan S. Deming C. Meyer J. A.. (2013). Topographic diversity of fungal and bacterial communities in human skin. Nature 498, 367–370. 10.1038/nature12171 23698366 PMC3711185

[B55] Finkbeiner S. R. Li Y. Ruone S. Conrardy C. Gregoricus N. Toney D.. (2009). Identification of a novel astrovirus (Astrovirus VA1) associated with an outbreak of acute gastroenteritis. J. Virol. 83, 10836–10839. 10.1128/JVI.00998-09 19706703 PMC2753140

[B56] Fiore A. E. (2004). Hepatitis A transmitted by food. Clin. Infect. Dis. 38, 705–715. 10.1086/381671 14986256

[B57] Forbes J. D. Knox N. C. Ronholm J. Pagotto F. Reimer A. (2017). Metagenomics: the next culture-independent game changer. Front. Microbiol. 8:1069. 10.3389/fmicb.2017.01069 28725217 PMC5495826

[B58] Foth B. J. Tsai I. J. Reid A. J. Bancroft A. J. Nichol S. Tracey A.. (2014). Whipworm genome and dual-species transcriptome analyses provide molecular insights into an intimate host-parasite interaction. Nat. Genet. 46, 693–700. 10.1038/ng.3010 24929830 PMC5012510

[B59] Franz E. Gras L. Dallman T. (2016). Significance of whole genome sequencing for surveillance, source attribution and microbial risk assessment of foodborne pathogens. Curr. Opin. Food Sci. 8, 74–79. 10.1016/j.cofs.2016.04.004

[B60] Galbraith E. A. Antonopoulos D. A. White B. A. (2004). Suppressive subtractive hybridization as a tool for identifying genetic diversity in an environmental metagenome: the rumen as a model. Environ. Microbiol., 6, 928–937. 10.1111/j.1462-2920.2004.00575.x 15305918

[B61] Ganova-Raeva L. Punkova L. Campo D. S. Dimitrova Z. Skums P. Vu N. H.. (2015). Cryptic hepatitis B and E in patients with acute hepatitis of unknown etiology. J. Infect. Dis. 212, 1962–1969. 10.1093/infdis/jiv315 26155829

[B62] Gargis A. S. Kalman L. Berry M. W. Bick D. P. Dimmock D. P. Hambuch T.. (2012). Assuring the quality of next-generation sequencing in clinical laboratory practice. Nat. Biotechnol. 30, 1033–1036. 10.1038/nbt.2403 23138292 PMC3827024

[B63] Gargis A. S. Kalman L. Bick D. P. da Silva C. Dimmock D. P. Funke B. H.. (2015). Good laboratory practice for clinical next-generation sequencing informatics pipelines. Nat. Biotechnol. 33, 689–693. 10.1038/nbt.3237 26154004 PMC6504172

[B64] Gillings M. R. Paulsen I. T. Tetu S. G. (2015). Ecology and evolution of the human microbiota: fire, farming and antibiotics. Genes 6, 841–857. 10.3390/genes6030841 26371047 PMC4584332

[B65] Gilmour M. W. Graham M. Van Domselaar G. Tyler S. Kent H. Trout-Yakel K. M.. (2010). High-throughput genome sequencing of two *Listeria monocytogenes* clinical isolates during a large foodborne outbreak. BMC Genomics 11:120. 10.1186/1471-2164-11-120 20167121 PMC2834635

[B66] GMI (2011). Perspectives of a Global, Real-Time Microbiological Genomic Identification System - Implications for National and Global Detection and Control of Infectious Diseases - Consensus Report of an Expert Meeting 1-2 September 2011, Bruxelles, Belgium: Global Microbial Identifier website.

[B67] GMI (2015). Protocol for GMI Proficiency Test, 2015 (Global Microbial Identifier). Available online at: http://www.globalmicrobialidentifier.org/-/media/Sites/gmi/Work-groups/GMI_PT_Protocol_v2_Incl_Appendices_2015_final_24082015.ashx?la=da.

[B68] Gomi R. Matsuda T. Matsui Y. Yoneda M. (2014). Fecal source tracking in water by next-generation sequencing technologies using host-specific *Escherichia coli* genetic markers. Environ. Sci. Technol. 48, 9616–9623. 10.1021/es501944c 25055157

[B69] Goodwin S. Gurtowski J. Ethe-Sayers S. Deshpande P. Schatz M. C. McCombie W. R. (2015). Oxford Nanopore sequencing, hybrid error correction, and de novo assembly of a eukaryotic genome. Genome Res. 25, 1750–1756. 10.1101/gr.191395.115 26447147 PMC4617970

[B70] Goodwin S. McPherson J. D. McCombie W. R. (2016). Coming of age: ten years of next-generation sequencing technologies. Nat. Rev. Genet. 17, 333–351. 10.1038/nrg.2016.49 27184599 PMC10373632

[B71] Greninger A. L. Naccache S. N. Federman S. Yu G. Mbala P. Bres V.. (2015). Rapid metagenomic identification of viral pathogens in clinical samples by real-time nanopore sequencing analysis. Genome Med. 7:99. 10.1186/s13073-015-0220-9 26416663 PMC4587849

[B72] Gurgui M. Sanchez F. March F. Lopez-Contreras J. Martino R. Cotura A.. (2011). Nosocomial outbreak of *Blastoschizomyces capitatus* associated with contaminated milk in a haematological unit. J. Hosp. Infect. 78, 274–278. 10.1016/j.jhin.2011.01.027 21658800

[B73] Gweon H. S. Oliver A. Taylor J. Booth T. Gibbs M. Read D. S.. (2015). PIPITS: an automated pipeline for analyses of fungal internal transcribed spacer sequences from the Illumina sequencing platform. Methods Ecol. Evol. 6, 973–980. 10.1111/2041-210X.12399 27570615 PMC4981123

[B74] Hadfield S. J. Pachebat J. A. Swain M. T. Robinson G. Cameron S. J. S. Alexander J.. (2015). Generation of whole genome sequences of new *Cryptosporidium hominis* and *Cryptosporidium parvum* isolates directly from stool samples. BMC Genomics 16:650. 10.1186/s12864-015-1805-9 26318339 PMC4552982

[B75] Hall A. J. (2012). Noroviruses: the Perfect Human Pathogens? J. Infect. Dis. 205, 1622–1624. 10.1093/infdis/jis251 22573872 PMC4699433

[B76] Halliday M. L. Kang L. Y. Zhou T. K. Hu M. D. Pan Q. C. Fu T. Y.. (1991). An epidemic of hepatitis-A attributable to the ingestion of raw clams in Shanghai, China. J. Infect. Dis. 164, 852–859. 10.1093/infdis/164.5.852 1658157

[B77] Hartmann E. M. Halden R. U. (2012). Analytical methods for the detection of viruses in food by example of CCL-3 bioagents. Anal. Bioanal. Chem. 404, 2527–2537. 10.1007/s00216-012-5974-y 22526652

[B78] Hasman H. Saputra D. Sicheritz-Ponten T. Lund O. Svendsen C. A. Frimodt-Möller N.. (2014). Rapid whole-genome sequencing for detection and characterization of microorganisms directly from clinical samples. J. Clin. Microbiol. 52, 139–146. 10.1128/JCM.02452-13 24172157 PMC3911411

[B79] Hayes S. Mahony J. Nauta A. van Sinderen D. (2017). Metagenomic approaches to assess bacteriophages in various environmental niches. Viruses 9:127. 10.3390/v9060127 28538703 PMC5490804

[B80] Hedberg C. W. Osterholm M. T. (1993). Outbreaks of food-borne and waterborne viral gastroenteritis. Clin. Microbiol. Rev. 6, 199–210. 10.1128/CMR.6.3.199 8395330 PMC358282

[B81] Henao O. L. Jones T. F. Vugia D. J. Griffin P. M. Network F. D. A. S. (2015). Foodborne diseases active surveillance network - 2 decades of achievements, 1996-2015. Emerging Infect. Dis. 21, 1529–1536. 10.3201/eid2109.150581 26292181 PMC4550136

[B82] Holland J. Spindler K. Horodyski F. Grabau E. Nichol S. VandePol S. (1982). Rapid evolution of RNA genomes. Science 215, 1577–1585. 10.1126/science.7041255 7041255

[B83] Holmes A. Allison L. Ward M. Dallman T. J. Clark R. Fawkes A.. (2015). Utility of whole-genome sequencing of *Escherichia coli* O157 for outbreak detection and epidemiological surveillance. J. Clin. Microbiol. 53, 3565–3573. 10.1128/JCM.01066-15 26354815 PMC4609728

[B84] Holst-Jensen A. Johannessen G. Sekse C. Spilsberg B. Dobnik D. Dreo T. (2016). Minimum Performance Parameters for Molecular Analytical Methods - Deliverable 6.6 of the Decathlon Project. Available online at: http://www.decathlon-project.eu/reports-and-deliverables

[B85] Holst-Jensen A. Rønning S. B. Løvseth A. Berdal K. G. (2003). PCR technology for screening and quantification of genetically modified organisms (GMOs). Anal. Bioanal. Chem. 375, 985–993. 10.1007/s00216-003-1767-7 12733008

[B86] Imamura S. Haruna M. Goshima T. Kanezashi H. Okada T. Akimoto K. (2016a). Application of next-generation sequencing to evaluate the profile of noroviruses in pre- and post-depurated oysters. Foodborne Pathog. Dis. 13, 559–565. 10.1089/fpd.2016.2150 27479133

[B87] Imamura S. Haruna M. Goshima T. Kanezashi H. Okada T. Akimoto K. (2016b). Application of next-generation sequencing to investigation of norovirus diversity in shellfish collected from two coastal sites in Japan from 2013 to (2014). Japan. J. Veterin. Res. 64, 113–122. 10.14943/jjvr.64.2.113 27506085

[B88] Iriart X. Fior A. Blanchet D. Berry A. Neron P. Aznar C. (2010). *Monascus ruber*: invasive gastric infection caused by dried and salted fish consumption. J. Clin. Microbiol. 48, 3800–3802. 10.1128/JCM.01000-10 20686087 PMC2953137

[B89] Jarvis K. G. White J. R. Grim C. J. Ewing L. Ottesen A. R. Beaubrun J. J.-G.. (2015). Cilantro microbiome before and after nonselective pre-enrichment for *Salmonella* using 16S rRNA and metagenomic sequencing. BMC Microbiol. 15:160. 10.1186/s12866-015-0497-2 26264042 PMC4534111

[B90] Jenkins C. Dallman T. J. Launders N. Willis C. Byrne L. Jorgensen F.. (2015). Public health investigation of two outbreaks of Shiga toxin-producing *Escherichia coli* O157 associated with consumption of watercress. Appl. Environ. Microbiol. 81, 3946–3952. 10.1128/AEM.04188-14 25841005 PMC4524134

[B91] Joensen K. G. Scheutz F. Lund O. Hasman H. Kaas R. S. Nielsen E. M.. (2014). Real-time whole-genome sequencing for routine typing, surveillance, and outbreak detection of verotoxigenic *Escherichia coli*. J. Clin. Microbiol. 52, 1501–1510. 10.1128/JCM.03617-13 24574290 PMC3993690

[B92] Jones Y. L. Peters S. M. Weland C. Ivanova N. V. Yancy H. F. (2013). Potential use of DNA barcodes in regulatory science: identification of the US food and drug administration's “Dirty 22,” contributors to the spread of foodborne pathogens. J. Food Prot. 76, 144–149. 10.4315/0362-028X.JFP-12-168 23317871

[B93] Junemann S. Sedlazeck F. J. Prior K. Albersmeier A. John U. Kalinowski J.. (2013). Updating benchtop sequencing performance comparison. Nat. Biotechnol. 31, 294–296. 10.1038/nbt.2522 23563421

[B94] Kawai T. Sekizuka T. Yahata Y. Kuroda M. Kumeda Y. Iijima Y.. (2012). Identification of *Kudoa septempunctata* as the causative agent of novel food poisoning outbreaks in Japan by consumption of *Paralichthys olivaceus* in raw fish. Clin. Infect. Dis. 54, 1046–1052. 10.1093/cid/cir1040 22281845

[B95] Kazan E. Maertens J. Herbrecht R. Weisser M. Gachot B. Vekhoff A.. (2011). A retrospective series of gut aspergillosis in haematology patients. Clin. Microbiol. Infect. 17, 588–594. 10.1111/j.1469-0691.2010.03310.x 20636423

[B96] Kergourlay G. Taminiau B. Daube G. Champomier Verges M.-C. (2015). Metagenomic insights into the dynamics of microbial communities in food. Int. J. Food Microbiol. 213, 31–39. 10.1016/j.ijfoodmicro.2015.09.010 26414193

[B97] Kundu S. Lockwood J. Depledge D. P. Chaudhry Y. Aston A. Rao K.. (2013). Next-generation whole genome sequencing identifies the direction of norovirus transmission in linked patients. Clin. Infect. Dis. 57, 407–414. 10.1093/cid/cit287 23645848 PMC3703108

[B98] Kupferschmidt K. (2011). Scientists rush to study genome of lethal *E. coli*. Science 332, 1249–1250. 10.1126/science.332.6035.1249 21659576

[B99] Kwon T. Yoo W. G. Lee W.-J. Kim W. Kim D.-W. (2015). Next-generation sequencing data analysis on cloud computing. Genes Genomics 37, 489–501. 10.1007/s13258-015-0280-7

[B100] Lambert D. Pightling A. Griffiths E. Van Domselaar G. Evans P. Berthelet S.. (2017). Baseline practices for the application of genomic data supporting regulatory food safety. J. AOAC Int. 100, 721–731. 10.5740/jaoacint.16-0269 28105974

[B101] Lammers Y. Peelen T. Vos R. A. Gravendeel B. (2014). The HTS barcode checker pipeline, a tool for automated detection of illegally traded species from high-throughput sequencing data. BMC Bioinformatics 15:44. 10.1186/1471-2105-15-44 24502833 PMC3922334

[B102] Lassen S. G. Ethelberg S. Bjorkman J. T. Jensen T. Sorensen G. Jensen A. K. (2016). Two listeria outbreaks caused by smoked fish consumption-using whole-genome sequencing for outbreak investigations. Clin. Microbiol. Infect. 21, 620–624. 10.1016/j.cmi.2016.04.017 27145209

[B103] Lassmann T. (2015). TagDust2: a generic method to extract reads from sequencing data. BMC Bioinformatics 16:24. 10.1186/s12859-015-0454-y 25627334 PMC4384298

[B104] Lee H. B. Patriarca A. Magan N. (2015). *Alternaria* in food: ecophysiology, mycotoxin production and toxicology. Mycobiology 43, 93–106. 10.5941/MYCO.2015.43.2.93 26190916 PMC4505009

[B105] Lee S. C. Billmyre R. B. Li A. Carson S. Sykes S. M. Huh E. Y.. (2014). Analysis of a food-borne fungal pathogen outbreak: virulence and genome of a *Mucor circinelloides* isolate from yogurt. mBio 5:e01390–14. 10.1128/mBio.01390-14 25006230 PMC4161253

[B106] Leekitcharoenphon P. Nielsen E. M. Kaas R. S. Lund O. Aarestrup F. M. (2014). Evaluation of whole genome sequencing for outbreak detection of *Salmonella enterica*. PLoS ONE 9:e87991. 10.1371/journal.pone.0087991 24505344 PMC3913712

[B107] Leichty A. R. Brisson D. (2014). Selective whole genome amplification for resequencing target microbial species from complex natural samples. Genetics 198, 473–481. 10.1534/genetics.114.165498 25096321 PMC4196606

[B108] Leonard S. R. Mammel M. K. Lacher D. W. Elkins C. A. (2015). Application of metagenomic sequencing to food safety: detection of Shiga toxin-producing *Escherichia coli* on fresh bagged spinach. Appl. Environ. Microbiol. 81, 8183–8191. 10.1128/AEM.02601-15 26386062 PMC4651076

[B109] Leonard S. R. Mammel M. K. Lacher D. W. Elkins C. A. (2016). Strain-level discrimination of Shiga toxin-producing *Escherichia coli* in spinach using metagenomic sequencing. PLoS ONE 11:e0167870. 10.1371/journal.pone.0167870 27930729 PMC5145215

[B110] Leray M. Knowlton N. (2015). DNA barcoding and metabarcoding of standardized samples reveal patterns of marine benthic diversity. Proc. Natl. Acad. Sci. U.S.A. 112, 2076–2081. 10.1073/pnas.1424997112 25646458 PMC4343139

[B111] Li J.-W. Schmieder R. Ward R. M. Delenick J. Olivares E. C. Mittelman D. (2012). SEQanswers: an open access community for collaboratively decoding genomes. Bioinformatics 28, 1272–1273. 10.1093/bioinformatics/bts128 22419780 PMC3338018

[B112] Lindsey R. L. Pouseele H. Chen J. C. Strockbine N. A. Carleton H. A. (2016). Implementation of Whole Genome Sequencing (WGS) for Identification and Characterization of Shiga Toxin-Producing *Escherichia coil* (STEC) in the United States. Front. Microbiol. 7:766 10.3389/fmicb.2016.00766 27242777 PMC4876609

[B113] Litvintseva A. P. Hurst S. Gade L. Frace M. A. Hilsabeck R. Schupp J. M.. (2014). Whole-genome analysis of *Exserohilum rostratum* from an outbreak of fungal meningitis and other infections. J. Clin. Microbiol. 52, 3216–3222. 10.1128/JCM.00936-14 24951807 PMC4313140

[B114] Litvintseva A. P. Marsden-Haug N. Hurst S. Hill H. Gade L. Driebe E. M.. (2015). Valley fever: finding new places for an old disease: *Coccidioides immitis* found in Washington State soil associated with recent human infection. Clin. Infect. Dis. 60, E1–E3. 10.1093/cid/ciu681 25165087 PMC4296125

[B115] Liu L. Li Y. Li S. Hu N. He Y. Pong R.. (2012). Comparison of next-generation sequencing systems. J. Biomed. Biotechnol. 2012:251364. 10.1155/2012/251364 22829749 PMC3398667

[B116] Livezey K. Kaplan S. Wisniewski M. Becker M. M. (2013). A new generation of food-borne pathogen detection based on ribosomal RNA. Annu. Rev. Food. Sci. Technol. 4, 313–325. 10.1146/annurev-food-050412-104448 23464575

[B117] Loman N. J. Constantinidou C. Christner M. Rohde H. Chan J. Z. M. Quick J.. (2013). A culture-independent sequence-based metagenomics approach to the investigation of an outbreak of Shiga-toxigenic *Escherichia coli* O104:H4. JAMA 309, 1502–1510. 10.1001/jama.2013.3231 23571589

[B118] Loman N. J. Misra R. V. Dallman T. J. Constantinidou C. Gharbia S. E. Wain J.. (2012). Performance comparison of benchtop high-throughput sequencing platforms. Nat. Biotechnol. 30:434. 10.1038/nbt0612-562f 22522955

[B119] Loman N. J. Pallen M. J. (2015). Twenty years of bacterial genome sequencing. Nat. Rev. Microbiol. 13, 787–794. 10.1038/nrmicro3565 26548914

[B120] Loman N. J. Quick J. Simpson J. T. (2015). A complete bacterial genome assembled *de novo* using only nanopore sequencing data. Nat. Methods 12, 733–U51. 10.1038/nmeth.3444 26076426

[B121] Loose M. Malla S. Stout M. (2016). Real-time selective sequencing using nanopore technology. Nat. Methods 13, 751–754. 10.1038/nmeth.3930 27454285 PMC5008457

[B122] Lukes J. Stensvold C. R. Jirku-Pomajbikova K. Parfrey L. W. (2015). Are human intestinal eukaryotes beneficial or commensals? PLoS Pathog. 11:e1005039 10.1371/journal.ppat.1005039 26270819 PMC4536199

[B123] Luo C. W. Tsementzi D. Kyrpides N. Read T. Konstantinidis K. T. (2012). Direct comparisons of Illumina vs. Roche 454 sequencing technologies on the same microbial community DNA sample. PLoS ONE 7:e30087. 10.1371/journal.pone.0030087 22347999 PMC3277595

[B124] Marchesi J. R. Ravel J. (2015). The vocabulary of microbiome research: a proposal. Microbiome 3:3. 10.1186/s40168-015-0094-5 26229597 PMC4520061

[B125] Martin J. Rosa B. A. Ozersky P. Hallsworth-Pepin K. Zhang X. Bhonagiri-Palsikar V.. (2015). Helminth.net: expansions to Nematode.net and an introduction to Trematode.net. Nucleic Acids Res. 43, D698–D706. 10.1093/nar/gku1128 25392426 PMC4383941

[B126] Martinovic T. Andjelkovic U. Gajdosik M. S. Resetar D. Josic D. (2016). Foodborne pathogens and their toxins. J. Proteomics 147, 226–235. 10.1016/j.jprot.2016.04.029 27109345

[B127] Mayo B. Rachid C. T. C. C. Alegria A. Leite A. M. O. Peixoto R. S. Delgado S. (2014). Impact of next generation sequencing techniques in food microbiology. Curr. Genomics 15, 293–309. 10.2174/1389202915666140616233211 25132799 PMC4133952

[B128] Mellmann A. Harmsen D. Cummings C. A. Zentz E. B. Leopold S. R. Rico A.. (2011). Prospective genomic characterization of the German enterohemorrhagic *Escherichia coli* O104:H4 outbreak by rapid next generation sequencing technology. PLoS ONE 6:e22751. 10.1371/journal.pone.0022751 21799941 PMC3140518

[B129] Menke S. Gillingham M. A. F. Wilhelm K. Sommer S. (2017). Home-made cost effective preservation buffer is a better alternative to commercial preservation methods for microbiome research. Front. Microbiol. 8:102. 10.3389/fmicb.2017.00102 28197142 PMC5281576

[B130] Mikheyev A. S. Tin M. M. (2014). A first look at the Oxford Nanopore MinION sequencer. Mol. Ecol. Resour. 14, 1097–1102. 10.1111/1755-0998.12324 25187008

[B131] Mizukoshi F. Kuroda M. Tsukagoshi H. Sekizuka T. Funatogawa K. Morita Y.. (2014). A food-borne outbreak of gastroenteritis due to genotype G1P[8] rotavirus among adolescents in Japan. Microbiol. Immunol. 58, 536–539. 10.1111/1348-0421.12176 25040046

[B132] Moore M. D. Goulter R. M. Jaykus L.-A. (2015). Human norovirus as a foodborne pathogen: challenges and developments. Annu. Rev. Food. Sci. Technol. 6, 411–433. 10.1146/annurev-food-022814-015643 25884284

[B133] Moran-Gilad J. Sintchenko V. Pedersen S. K. Wolfgang W. J. Pettengill J. Strain E.. (2015). Proficiency testing for bacterial whole genome sequencing: an end-user survey of current capabilities, requirements and priorities. BMC Infect. Dis. 15:174. 10.1186/s12879-015-0902-3 25887164 PMC4392855

[B134] Moreira D. López-García P. (2009). Ten reasons to exclude viruses from the tree of life. Nat. Rev. Microbiol. 7, 306–311. 10.1038/nrmicro2108 19270719

[B135] Moura A. Criscuolo A. Pouseele H. Maury M. Leclercq A. Tarr C. (2016). Whole genome-based population biology and epidemiological surveillance of Listeria monocytogenes. Nat. Microbiol. 10:16185 10.1038/nmicrobiol.2016.185 PMC8903085 27723724

[B136] Müenz M. Ruark E. Renwick A. Ramsay E. Clarke M. Mahamdallie S. (2015). CSN and CAVA: variant annotation tools for rapid, robust next-generation sequencing analysis in the clinical setting. Genome Med. 7:76 10.1186/s13073-015-0195-6 26315209 PMC4551696

[B137] Newell D. G. Koopmans M. Verhoef L. Duizer E. Aidara-Kane A. Sprong H.. (2010). Food-borne diseases - The challenges of 20 years ago still persist while new ones continue to emerge. Int. J. Food Microbiol. 139, S3–S15. 10.1016/j.ijfoodmicro.2010.01.021 20153070 PMC7132498

[B138] Nieuwenhuijse D. F. Koopmans M. P. G. (2017). Metagenomic sequencing for surveillance of food- and waterborne viral diseases. Front. Microbiol. 8:230. 10.3389/fmicb.2017.00230 28261185 PMC5309255

[B139] Octavia S. Wang Q. Tanaka M. M. Kaur S. Sintchenko V. Lan R. (2015). Delineating community outbreaks of *Salmonella enterica* serovar Typhimurium by use of whole-genome sequencing: insights into genomic variability within an outbreak. J. Clin. Microbiol. 53, 1063–1071. 10.1128/JCM.03235-14 25609719 PMC4365247

[B140] OIE (2017). Manual of Diagnostic Tests and Vaccines for Terrestrial Animals. Paris: World Organisation for Animal Health.

[B141] Olsen A. R. Gecan J. S. Ziobro G. C. Bryce J. R. (2001). Regulatory action criteria for filth and other extraneous materials V. Strategy for evaluating hazardous and nonhazardous filth. Regul. Toxicol. Pharmacol. 33, 363–392. 10.1006/rtph.2001.1472 11407939

[B142] Ondov B. D. Treangen T. J. Melsted P. Mallonee A. B. Bergman N. H. Koren S.. (2016). Mash: fast genome and metagenome distance estimation using MinHash. Genome Biol. 17:132. 10.1186/s13059-016-0997-x 27323842 PMC4915045

[B143] Orlandi P. A. Chu D. M. T. Bier J. W. Jackson G. J. (2002). Parasites and the food supply. Food Technol. 56, 72–81. Available online at: http://www.ift.org/~/media/Knowledge%20Center/Science%20Reports/Scientific%20Status%20Summaries/parasitesfoodsupply_0402.pdf

[B144] Ottesen A. Ramachandran P. Reed E. White J. R. Hasan N. Subramanian P.. (2016). Enrichment dynamics of Listeria monocytogenes and the associated microbiome from naturally contaminated ice cream linked to a listeriosis outbreak. BMC Microbiol. 16:275. 10.1186/s12866-016-0894-1 27852235 PMC5112668

[B145] Ottesen A. R. Gonzalez A. Bell R. Arce C. Rideout S. Allard M.. (2013). Co-enriching microflora associated with culture based methods to detect *Salmonella* from tomato phyllosphere. PLoS ONE 8:e73079. 10.1371/journal.pone.0073079 24039862 PMC3767688

[B146] Petersen T. N. Rasmussen S. Hasman H. Caroe C. Baelum J. Schultz A. C. (2015). Meta-genomic analysis of toilet waste from long distance flights; a step towards global surveillance of infectious diseases and antimicrobial resistance. Sci. Rep. 5:11444 10.1038/srep11444 26161690 PMC4498435

[B147] Pipan V. Kunaj T. (2015). Initiative for standardization of the format of the next generation sequencing (NGS) results. Discoveries 3:4 10.15190/d.2015.36 PMC6941547 32309567

[B148] Pires S. M. Fischer-Walker C. L. Lanata C. F. Devleesschauwer B. Hall A. J. Kirk M. D.. (2015). Aetiology-specific estimates of the global and regional incidence and mortality of diarrhoeal diseases commonly transmitted through food. PLoS ONE 10:e0142927. 10.1371/journal.pone.0142927 26632843 PMC4668836

[B149] Pires S. M. Vieira A. R. Perez E. Wong D. L. F. Hald T. (2012). Attributing human foodborne illness to food sources and water in Latin America and the Caribbean using data from outbreak investigations. Int. J. Food Microbiol. 152, 129–138. 10.1016/j.ijfoodmicro.2011.04.018 21570732

[B150] Quick J. Ashton P. Calus S. Chatt C. Gossain S. Hawker J.. (2015). Rapid draft sequencing and real-time nanopore sequencing in a hospital outbreak of *Salmonella*. Genome Biol. 16:114. 10.1186/s13059-015-0677-2 26025440 PMC4702336

[B151] Quick J. Grubaugh N. D. Pullan S. T. Claro I. M. Smith A. D. Gangavarapu K.. (2017). Multiplex PCR method for MinION and Illumina sequencing of Zika and other virus genomes directly from clinical samples. Nat. Protoc. 12, 1261–1276. 10.1038/nprot.2017.066 28538739 PMC5902022

[B152] Quick J. Loman N. J. Duraffour S. Simpson J. T. Ettore S. Cowley L.. (2016). Real-time, portable genome sequencing for Ebola surveillance. Nature 530, 228-+. 10.1038/nature16996 26840485 PMC4817224

[B153] Ranjan R. Rani A. Metwally A. McGee H. S. Perkins D. L. (2016). Analysis of the microbiome: advantages of whole genome shotgun versus 16S amplicon sequencing. Biochem. Biophys. Res. Commun. 469, 967–977. 10.1016/j.bbrc.2015.12.083 26718401 PMC4830092

[B154] Rasko D. A. Webster D. R. Sahl J. W. Bashir A. Boisen N. Scheutz F.. (2011). Origins of the *E. coli* strain causing an outbreak of hemolytic-uremic syndrome in Germany. New Engl. J. Med. 365, 709–717. 10.1056/NEJMoa1106920 21793740 PMC3168948

[B155] Ratan A. Olson T. L. Loughran T. P. Jr. Miller W. (2015). Identification of indels in next-generation sequencing data. BMC Bioinformatics 16:42. 10.1186/s12859-015-0483-6 25879703 PMC4339746

[B156] Reuter S. Ellington M. J. Cartwright E. J. P. Köser C. U. Török M. E. Gouliouris T.. (2013). Rapid bacterial whole-genome sequencing to enhance diagnostic and public health microbiology. JAMA Intern. Med. 173, 1397–1404. 10.1001/jamainternmed.2013.7734 23857503 PMC4001082

[B157] Rhoads A. Au K. F. (2015). PacBio sequencing and its applications. Genom. Proteom. Bioinform. 13, 278–289. 10.1016/j.gpb.2015.08.002 26542840 PMC4678779

[B158] Robertson L. J. Sprong H. Ortega Y. R. van der Giessen J. W. B. Fayer R. (2014). Impacts of globalisation on foodborne parasites. Trends Parasitol. 30, 37–52. 10.1016/j.pt.2013.09.005 24140284

[B159] Roden M. M. Zaoutis T. E. Buchanan W. L. Knudsen T. A. Sarkisova T. A. Schaufele R. L.. (2005). Epidemiology and outcome of zygomycosis: a review of 929 reported cases. Clin. Infect. Dis. 41, 634–653. 10.1086/432579 16080086

[B160] Rodríguez-Lazaro D. Cook N. Ruggeri F. M. Sellwood J. Nasser A. Nascimento M. S. J.. (2012). Virus hazards from food, water and other contaminated environments. FEMS Microbiol. Rev. 36, 786–814. 10.1111/j.1574-6976.2011.00306.x 22091646 PMC7114518

[B161] Salmond G. P. C. Fineran P. C. (2015). A century of the phage: past, present and future. Nat. Rev. Microbiol. 13, 777–786. 10.1038/nrmicro3564 26548913

[B162] Sboner A. Mu X. J. Greenbaum D. Auerbach R. K. Gerstein M. B. (2011). The real cost of sequencing: higher than you think! Genome Biol. 12:125. 10.1186/gb-2011-12-8-125 21867570 PMC3245608

[B163] Scallan E. Hoekstra R. M. Angulo F. J. Tauxe R. V. Widdowson M.-A. Roy S. L.. (2011). Foodborne illness acquired in the United States - major pathogens. Emerging Infect. Dis. 17, 7–15. 10.3201/eid1701.P11101 21192848 PMC3375761

[B164] Scheutz F. Nielsen E. M. Frimodt-Möller J. Boisen N. Morabito S. Tozzoli R.. (2011). Characteristics of the enteroaggregative Shiga toxin/verotoxin-producing *Escherichia coli* O104:H4 strain causing the outbreak of haemolytic uraemic syndrome in Germany, May to June (2011). Eurosurveillance 16, 5–10. 10.2807/ese.16.24.19889-en 21699770

[B165] Schmid D. Allerberger F. Huhulescu S. Pietzka A. Amar C. Kleta S.. (2014). Whole genome sequencing as a tool to investigate a cluster of seven cases of listeriosis in Austria and Germany, 2011-2013. Clin. Microbiol. Infect. 20, 431–436. 10.1111/1469-0691.12638 24698214 PMC4232032

[B166] Schmitz-Esser S. Wagner M. (2014). Genome sequencing of *Listeria monocytogenes*. Methods Mol. Biol. 1157, 223–232. 10.1007/978-1-4939-0703-8_19 24792562

[B167] Severi E. Verhoef L. Thornton L. Guzman-Herrador B. R. Faber M. Sundqvist L.. (2015). Large and prolonged food-borne multistate hepatitis A outbreak in Europe associated with consumption of frozen berries, 2013 to (2014). Eurosurveillance 20, 11–19. 10.2807/1560-7917.ES2015.20.29.21192 26227370

[B168] Singer E. Bushnell B. Coleman-Derr D. Bowman B. Bowers R. M. Levy A.. (2016). High-resolution phylogenetic microbial community profiling. ISME J. 10, 2020–2032. 10.1038/ismej.2015.249 26859772 PMC5029162

[B169] Smits S. L. Schapendonk C. M. E. van Beek J. Vennema H. Schürch A. C. Schipper D.. (2014). New viruses in idiopathic human diarrhea cases, the Netherlands. Emerging Infect. Dis. 20, 1218–1222. 10.3201/eid2007.140190 24964003 PMC4073879

[B170] Spellberg B. (2012). Gastrointestinal mucormycosis: an evolving disease. Gastroenterol. Hepatol. 8, 140–142. 22485085 PMC3317515

[B171] Spilsberg B. Lagesen K. Kristoffersen A. B. Holst-Jensen A. (2017). Identification and quantification of genetically modified organisms (GMO) from high throughput sequencing data, in qPCR dPCR & NGS (2017). eds Bustin S. Pfaffl M. W. (Freising: Biomolecular Detection and Quantification), 11, S33.

[B172] Staats M. Arulandhu A. J. Gravendeel B. Holst-Jensen A. Scholtens I. Peelen T.. (2016). Advances in DNA metabarcoding for food and wildlife forensic species identification. Anal. Bioanal. Chem. 408, 4615–4630. 10.1007/s00216-016-9595-8 27178552 PMC4909793

[B173] Stapleton A. E. (2014). A biologist, a statistician, and a bioinformatician walk into a conference room…and walk out with a great metagenonnics project plan. Front. Plant Sci. 5:250 10.3389/fpls.2014.00250 24917875 PMC4042100

[B174] Stasiewicz M. J. Oliver H. F. Wiedmann M. den Bakker H. C. (2015). Whole-genome sequencing allows for improved identification of persistent *Listeria monocytogenes* in food-associated environments. Appl. Environ. Microbiol. 81, 6024–6037. 10.1128/AEM.01049-15 26116683 PMC4551262

[B175] Stoev S. D. (2015). Foodborne mycotoxicoses, risk assessment and underestimated hazard of masked mycotoxins and joint mycotoxin effects or interaction. Environ. Toxicol. Pharmacol. 39, 794–809. 10.1016/j.etap.2015.01.022 25734690

[B176] Struelens M. J. Palm D. Takkinen J. (2011). Enteroaggregative, Shiga toxin-producing *Escherichia coli* O104:H4 outbreak: new microbiological findings boost coordinated investigations by European public health laboratories. Eurosurveillance 16, 2–4. 10.2807/ese.16.24.19890-en 21699771

[B177] Taboada E. N. Graham M. R. Carriço J. A. Van Domselaar G. (2017). Food safety in the age of next generation sequencing, bioinformatics, and open data access. Front. Microbiol. 8:909. 10.3389/fmicb.2017.00909 28588568 PMC5440521

[B178] Tallon L. J. Liu X. Bennuru S. Chibucos M. C. Godinez A. Ott S.. (2014). Single molecule sequencing and genome assembly of a clinical specimen of *Loa loa*, the causative agent of loiasis. BMC Genomics 15:788. 10.1186/1471-2164-15-788 25217238 PMC4175631

[B179] Tan B. Ng C. Nshimyimana J. P. Loh L. L. Gin K. Y. H. Thompson J. R. (2015). Next-generation sequencing (NGS) for assessment of microbial water quality: current progress, challenges, and future opportunities. Front. Microbiol. 6:1027. 10.3389/fmicb.2015.01027 26441948 PMC4585245

[B180] Taylor A. J. Lappi V. Wolfgang W. J. Lapierre P. Palumbo M. J. Medus C.. (2015). Characterization of foodborne outbreaks of *Salmonella enterica* serovar Enteritidis with whole-genome sequencing single nucleotide polymorphism-based analysis for surveillance and outbreak detection. J. Clin. Microbiol. 53, 3334–3340. 10.1128/JCM.01280-15 26269623 PMC4572550

[B181] Tilden J. Young W. McNamara A. M. Custer C. Boesel B. LambertFair M.. (1996). A new route of transmission for *Escherichia coli*: infection from dry fermented salami. Am. J. Public Health 86, 1142–1145. 10.2105/AJPH.86.8_Pt_1.1142 8712275 PMC1380627

[B182] Timme R. Rand H. Trees E. Agarwala R. David S. Shumway M. (2015). Benchmark datasets for validating foodborne outbreak investigations: integrating WGS and phylogenomic analyses, in 1st ASM Conference on Rapid Next-Generation Sequencing and Bioinformatic Pipelines for Enhanced Molecular Epidemiologic Investigation of Pathogens (Washington, DC).

[B183] Timme R. E. Allard M. W. Luo Y. Strain E. Pettengill J. Wang C.. (2012). Draft genome sequences of 21 *Salmonella enterica* serovar Enteritidis strains. J. Bacteriol. 194, 5994–5995. 10.1128/JB.01289-12 23045502 PMC3486122

[B184] Tsai I. J. Zarowiecki M. Holroyd N. Garciarrubio A. Sanchez-Flores A. Brooks K. L.. (2013). The genomes of four tapeworm species reveal adaptations to parasitism. Nature 496, 57–63. 10.1038/nature12031 23485966 PMC3964345

[B185] Turabelidze G. Lawrence S. J. Gao H. Sodergren E. Weinstock G. M. Abubucker S.. (2013). Precise dissection of an *Escherichia coli* O157:H7 outbreak by single nucleotide polymorphism analysis. J. Clin. Microbiol. 51, 3950–3954. 10.1128/JCM.01930-13 24048526 PMC3838074

[B186] Tuttle J. Gomez T. Doyle M. P. Wells J. G. Zhao T. Tauxe R. V.. (1999). Lessons from a large outbreak of *Escherichia coli* O157: H7 infections: insights into the infectious dose and method of widespread contamination of hamburger patties. Epidemiol. Infect. 122, 185–192. 10.1017/S0950268898001976 10355781 PMC2809605

[B187] Underwood A. P. Dallman T. Thomson N. R. Williams M. Harker K. Perry N.. (2013). Public health value of next-generation DNA sequencing of enterohemorrhagic *Escherichia coli* isolates from an outbreak. J. Clin. Microbiol. 51, 232–237. 10.1128/JCM.01696-12 23135946 PMC3536255

[B188] van Dijk E. L. Auger H. Jaszczyszyn Y. Thermes C. (2014). Ten years of next-generation sequencing technology. Trends Genet. 30, 418–426. 10.1016/j.tig.2014.07.001 25108476

[B189] Vaux S. Criscuolo A. Desnos-Ollivier M. Diancourt L. Tarnaud C. Vandenbogaert M.. (2014). Multicenter outbreak of infections by *Saprochaete clavata*, an unrecognized opportunistic fungal pathogen. mBio 5:e02309–14. 10.1128/mBio.02309-14 25516620 PMC4271555

[B190] Wang B. Cunningham J. M. Yang X. (2015a). Seq2pathway: an R/Bioconductor package for pathway analysis of next-generation sequencing data. Bioinformatics 31, 3043–3045. 10.1093/bioinformatics/btv289 25979472 PMC4565027

[B191] Wang C. Zhang D. (2011). A novel compression tool for efficient storage of genome resequencing data. Nucleic Acids Res. 39, E45–U74. 10.1093/nar/gkr009 21266471 PMC3074166

[B192] Wang Q. Holmes N. Martinez E. Howard P. Hill-Cawthorne G. Sintchenko V. (2015b). It is not all about single nucleotide polymorphisms: comparison of mobile genetic elements and deletions in *Listeria monocytogenes* genomes links cases of hospital-acquired listeriosis to the environmental source. J. Clin. Microbiol. 53, 3492–3500. 10.1128/JCM.00202-15 26311854 PMC4609684

[B193] Wang S.-H. Shen M. Lin H.-C. Sun P.-L. Lo H.-J. Lu J.-J. (2015c). Molecular epidemiology of invasive *Candida albicans* at a tertiary hospital in northern Taiwan from 2003 to (2011). Med. Mycol. 53, 828–836. 10.1093/mmy/myv065 26333357

[B194] Wang Y. Ye Z. Ying Y. (2012). New trends in impedimetric biosensors for the detection of foodborne pathogenic bacteria. Sensors 12, 3449–3471. 10.3390/s120303449 22737018 PMC3376556

[B195] Weinmaier T. Probst A. J. La Duc M. T. Ciobanu D. Cheng J. F. Ivanova N.. (2015). A viability-linked metagenomic analysis of cleanroom environments: eukarya, prokaryotes, and viruses. Microbiome 3:62. 10.1186/s40168-015-0129-y 26642878 PMC4672508

[B196] Weiss M. M. Van der Zwaag B. Jongbloed J. D. H. Vogel M. J. Brüggenwirth H. T. Deprez R. H. L.. (2013). Best practice guidelines for the use of next-generation sequencing applications in genome diagnostics: a national collaborative study of Dutch genome diagnostic laboratories. Hum. Mutat. 34, 1313–1321. 10.1002/humu.22368 23776008

[B197] Withlow L. Hagler W. Jr. (2016). Mold and Mycotoxin Issues in Dairy Cattle: Effects, Prevention and Treatment. EXTENSION [Online]. Available online at: http://articles.extension.org/pages/11768/mold-and-mycotoxin-issues-in-dairy-cattle:-effects-prevention-and-treatment

[B198] Wong T. H. N. Dearlove B. L. Hedge J. Giess A. P. Piazza P. Trebes A.. (2013). Whole genome sequencing and *de novo* assembly identifies Sydney-like variant noroviruses and recombinants during the winter 2012/2013 outbreak in England. Virol. J. 10:335. 10.1186/1743-422X-10-335 24220146 PMC3874643

[B199] Wu Y. Zheng J. Wang Y. Li S. Jin H. Li Z.. (2015). Draft genome sequence of *Listeria monocytogenes* LM201, isolated from foodstuff. Genome Announce. 3:e01417–14. 10.1128/genomeA.01417-14 25657267 PMC4319610

[B200] Wuyts V. Denayer S. Roosens N. H. C. Mattheus W. Bertrand S. Marchal K. (2015). Whole genome sequence analysis of *Salmonella* Enteritidis PT4 outbreaks from a national reference laboratory's viewpoint. PLoS Curr. Outbreaks 2015:1 10.1371/currents.outbreaks.aa5372d90826e6cb0136ff66bb7a62fc PMC4593640 26468422

[B201] Xia J. Wang Q. Jia P. Wang B. Pao W. Zhao Z. (2012). NGS Catalog: a database of next generation sequencing studies in humans. Hum. Mutat. 33, E2341–E2355. 10.1002/humu.22096 22517761 PMC4431973

[B202] Young N. D. Nagarajan N. Lin S. J. Korhonen P. K. Jex A. R. Hall R. S.. (2014). The *Opisthorchis viverrini* genome provides insights into life in the bile duct. Nat. Commun. 5:4378. 10.1038/ncomms5378 25007141 PMC4104445

[B203] Zhang S. K. Yin Y. L. Jones M. B. Zhang Z. Z. Kaiser B. L. D. Dinsmore B. A.. (2015). *Salmonella* serotype determination utilizing high-throughput genome sequencing data. J. Clin. Microbiol. 53, 1685–1692. 10.1128/JCM.00323-15 25762776 PMC4400759

